# Species delimitation using morphology, morphometrics, and molecules: definition of the *Ophion scutellaris* Thomson species group, with descriptions of six new species (Hymenoptera, Ichneumonidae)

**DOI:** 10.3897/zookeys.462.8229

**Published:** 2014-12-10

**Authors:** Marla D. Schwarzfeld, Felix A. H. Sperling

**Affiliations:** 1 Department of Biological Sciences, CW405, Biological Sciences Centre, University of Alberta, Edmonton, AB, T6G 2E9; 2 Ecosystem Science and Management Program, University of Northern British Columbia, 3333 University Way, Prince George, BC, V2N 4Z9

**Keywords:** Barcoding, geometric morphometrics, Ichneumonidae, integrative taxonomy, molecular taxonomy, *Ophion*, species delimitation

## Abstract

The diverse genus *Ophion* is almost entirely undescribed in the Nearctic region. In this paper we define the *Ophion scutellaris* species group. This species group is well-supported by analysis of DNA (ITS2, COI, and 28S D2-D3) and morphology. It includes the Palearctic species *Ophion scutellaris* and the Nearctic species *Ophion idoneus*. An integrative analysis of DNA, geometric wing morphometrics, classical morphometrics and qualitative morphology indicates that this species group contains a minimum of seven species in North America, although the full diversity of the group has likely not been sampled. *Ophion clave* Schwarzfeld, **sp. n.**, *Ophion aureus* Schwarzfeld, **sp. n.**, *Ophion brevipunctatus* Schwarzfeld, **sp. n.**, *Ophion dombroskii* Schwarzfeld, **sp. n.**, *Ophion keala* Schwarzfeld, **sp. n.** and *Ophion importunus* Schwarzfeld, **sp. n.** are described, and a key to the known Nearctic species of the *Ophion scutellaris* group is provided.

## Introduction

*Ophion* Fabricius is a genus of large nocturnal Ichneumonidae in the subfamily Ophioninae. Most species parasitize medium to large-sized Lepidoptera larvae, especially Noctuoidea (Townes 1971; Brock 1982). Whereas Ophioninae are generally more diverse in the tropics, *Ophion* is most diverse in temperate regions (Townes 1971; Gauld 1985). Gauld (1985) estimated that the Nearctic fauna consists of approximately 50 species, and molecular work suggests the number is much higher (Schwarzfeld and Sperling, in prep.). However only eleven Nearctic species of *Ophion* are currently described (Yu et al. 2012), the most recent of which were described a century ago (Hooker 1912; Morley 1912).

While several species groups of *Ophion* have been proposed (summarized by Gauld 1985), almost all Nearctic species were included within the “*Ophion luteus* species group”. Gauld (1985) acknowledged that this species group was paraphyletic and defined by plesiomorphies, but not enough was known about the Nearctic species to further subdivide it into monophyletic groupings.

*Ophion* species are difficult to distinguish morphologically and have a great deal of intraspecific variability (Townes 1971; Brock 1982). Molecular taxonomy has been proposed as a method to accurately delimit and identify species that lack useful morphological characters (Caterino et al. 2000; Tautz et al. 2003; Blaxter 2004). In particular, DNA barcoding, or the sequencing of a standardized 658 bp segment of the mitochondrial cytochrome c oxidase I gene (COI), has been promoted as a method for species discovery, identification and delimitation (Hebert et al. 2003). Among the advantages of COI are its rapid evolution (hence potentially informative at the species level) and presence in multiple copies (hence easily amplified). However, many studies have argued that COI alone is not sufficient to accurately delimit species due to factors such as hybridization, retained ancestral polymorphisms and high intraspecific variability (Funk and Omland 2003; Cognato 2006; Meier et al. 2006; Schmidt and Sperling 2008; Dupuis et al. 2012).

To avoid relying on a single mitochondrial gene, nuclear genes provide an additional data source to evaluate species boundaries (Simon et al. 2006). The internal transcribed spacer of nuclear ribosomal DNA (ITS2) is another rapidly evolving gene that has been successfully used for species delimitation in insects (e.g. Alvarez and Hoy 2002; Hung et al. 2004; Wagener et al. 2006). It too, however, can potentially give misleading results due to high intraspecific or even intra-individual variation (Rich et al. 1997; Harris and Crandall 2000; Li and Wilkerson 2007). It can also be difficult to align accurately due to the presence of numerous insertion-deletion events. A second nuclear gene, the nuclear ribosomal 28S gene is a highly conserved gene that has often been used for higher level insect phylogeny (Caterino et al. 2000), though it has also proved useful in distinguishing species (e.g. Monaghan et al. 2005; Derycke et al. 2008; Raupach et al. 2010). In particular, the D2-D3 expansion region of 28S rDNA is among the most commonly used molecular markers for Hymenoptera phylogenies (e.g. Mardulyn and Whitfield 1999; Dowton and Austin 2001; Laurenne et al. 2006; Quicke et al. 2009; Klopfstein et al. 2011).

Quantitative morphometric analyses have been shown to be at least as accurate as molecular analyses in the delimitation of morphologically challenging species, and possibly more so (Lumley and Sperling 2010). Classical (or traditional) morphometrics involves direct measurement of various characters that are then analyzed using multivariate methods (Marcus 1990; Mutanen and Pretorius 2007). This technique is often successful at separating similar species, even when there is overlap between the individual characters (e.g. Lumley and Sperling 2010; Buck et al. 2012). Alternatively, geometric morphometrics analyzes overall changes in shape by using landmarks of homologous structures (Rohlf and Marcus 1993). Insect wings are an ideal subject for geometric morphometric studies, as they are two-dimensional, easily imaged and the venation provides many points that are clearly homologous and straightforward to landmark accurately. Geometric morphometric analysis of wing venation has been used successfully to discriminate species of several complexes of closely related insect species (e.g. Villemant et al. 2007; Tofilski 2008; Francuski et al. 2009; Milankov et al. 2009), but to our knowledge has not yet been used for Ichneumonidae. Although not using geometric morphometrics, two studies show promise for separating closely related Ichneumonidae species based on overall wing venation (Yu et al. 1992; Weeks et al. 1997).

The use of multiple lines of evidence to circumscribe species is often referred to as “integrative taxonomy” (Dayrat 2005; Will et al. 2005; Sperling and Roe 2009; Schlick-Steiner et al. 2010; Dupuis et al. 2012). Our study more closely conforms to the concept of “iterative taxonomy” (Yeates et al. 2011), as we used morphology, multiple genes, geometric morphometrics and classical morphometrics to iteratively assess and evaluate species hypotheses. Specimens were first chosen for sequencing based on the identification of morphospecies. The molecular work identified the species group as a whole, and presented a framework for the relationships of taxa within the group. Putative species were then analyzed morphometrically and re-examined morphologically to assess the validity of the species hypotheses and to refine morphospecies concepts.

In this paper, we define the *Ophion scutellaris* species group, the first *Ophion* species group to be circumscribed based on both molecular and morphological characters. We then revise the known Nearctic species within this group, including descriptions of six new species.

## Methods

### Depositories of material examined

BMNH The Natural History Museum, London, UK

CNC Canadian National Collection of Insects, Arachnids and Nematodes, Ottawa, Ontario, Canada

CUIC Cornell University, Ithaca, New York, USA

NFRC Northern Forest Research Centre, Edmonton, Alberta, Canada

RBCM Royal British Columbia Museum, Victoria, British Columbia, Canada

SEMC Snow Entomological Museum, University of Kansas, Lawrence, Kansas, USA

SFUC Simon Fraser University, Burnaby, British Columbia, Canada

UASM E. H. Strickland Entomology Collection, University of Alberta, Edmonton, Alberta, Canada

UBCZ Spencer Entomology Collection, University of British Columbia, Vancouver, British Columbia, Canada

### Recognition of a putative species group and overall approach

This species group was first identified as part of a large-scale taxonomic and phylogenetic study of *Ophion* (Schwarzfeld et al., in prep.). From the sequencing results of several dozen *Ophion* morphospecies, a well-supported clade that included the Palearctic species *Ophion scutellaris* Thomson was identified (Schwarzfeld et al., in prep.). Morphological comparison showed that several of the characters that define *Ophion scutellaris*, according to Brock (1982), were characteristic of the group as a whole. Additional characters were recognized based on examination of all specimens recovered within this group. We named the group the *Ophion scutellaris* species group, after the oldest described species within the group, and used this suite of characters (defined in the Taxonomic Part, below) to locate all specimens within the group from available unsequenced material. We then used an iterative analysis of quantitative morphometrics, morphology, and molecular data to assess all sequenced and unsequenced specimens and to determine species boundaries within this newly-defined species group.

### Specimens and sampling

Most specimens were newly collected as part of a large-scale study of Canadian *Ophion*. Out of more than 4000 specimens that were collected from a range of habitats and localities across Canada (primarily from light-traps and Malaise traps), 196 were from the *Ophion scutellaris* species group. We also borrowed 662 specimens of *Ophion* from the following institutions: CUIC, UBCZ, SEMC, RBCM, CNC, NFRC, SFUC; these included 42 specimens from the species group. Five specimens of *Ophion scutellaris* were sequenced from British material provided by G. Broad (BMNH). We examined three of these, while for two specimens, we were sent legs for sequencing but have not seen the specimens.

### DNA sequencing

We sequenced one mitochondrial and two nuclear genes for the *Ophion scutellaris* group: the cytochrome c oxidase I gene (COI), the internal transcribed spacer 2 (ITS2), and the D2-D3 variable region of 28S rRNA. We also sequenced these three genes for eight species of *Ophion* outside of the target species group; these were chosen to represent the diversity across *Ophion*, based on a large-scale molecular analysis of *Ophion* (Schwarzfeld and Sperling, in prep.). Finally we sequenced one individual of *Enicospilus*, another genus within Ophioninae, as an outgroup. DNA was extracted from a single hind leg using DNeasy Blood & Tissue Kits (Qiagen, Toronto, ON); the final elution volume was 150 μL. We conducted PCR in either 50 μL or 15 μL reactions. The 50 μL reactions contained 4–8 μL genomic DNA, 5 μL 10× PCR buffer (containing 15 mmol/μL MgCl_2_) (Promega, Madison, WI), 3 μL of 25 mmoles/μL MgCl_2_ (Promega), 1 μL of 10 mmoles/μL dNTP’s (Roche, Switzerland), 1 μL each of 5pmol/μL forward and reverse primers, 0.5 μL of 5 U/μL *Taq* polymerase (Fermentation Service Unit, University of Alberta) and 30.5–34.5 μL of autoclaved Millipore water. The 15 μL reactions used 4–8 μL DNA, 1.5 μL PCR buffer, 0.9 μL MgCl_2_, 0.3 μL each of dNTP’s, forward and reverse primers, 0.15 μL *Taq* and 3.55–7.55 μL water (all concentrations as above). All PCR products were purified using ExoSap-IT (USB Corporation, Cleveland, OH), and were sequenced using BigDye Terminator version 3.1 cycle sequencing kit (Applied Biosystems, Foster City, CA), followed by ethanol precipitation. Sequencing reactions were run on an ABI Prism 3730 DNA analyser. Sequences are deposited in NCBI GenBank, and Genbank accession numbers are listed in Appendix 1.

To sequence COI, we used the primers lco hym (5’–CAA ATC ATA AAG ATA TTG G–3’) and hco out (5’–CCA GGT AAA ATT AAA ATA TAA ACT TC–3’) (Schulmeister 2003), which produce a fragment equivalent to the “barcode” region (Hebert et al. 2003); in *Ophion*, this region is 676 base pairs in length. PCR conditions were: 94° for 2 min, 35 cycles of 94° for 30 s, 45° for 30 s, 72° for 2 min, and a final extension at 72° for 5 min. Alignment was unambiguous, and was confirmed by translating nucleotides to amino acids in Mesquite (Maddison and Maddison 2011). We sequenced COI for 30 Nearctic and 5 Palearctic specimens within the *Ophion scutellaris* group (Appendix 1).

We analyzed ITS2 using the primers ITS2-F (5’-GGG TCG ATG AAG AAC GCA GC-3’) and ITS2-R (5’-ATA TGC TTA AAT TCA GCG GG-3’) which anneal to the flanking 28S and 5.8S genes (Navajas et al. 1994). PCR cycling was 94° for 2 min, 35 cycles of 94° for 30 s, 55° for 1 min, 72° for 2 min and a final extension of 75° for 5 min. Sequences were aligned using ClustalW (Larkin et al. 2007), and then were modified by eye in Mesquite. Large numbers of indels and highly divergent sequences made alignment of the *scutellaris* group with the non-*scutellaris* group sequences subjective; however within the *scutellaris* group the alignment was unambiguous. We were not able to successfully sequence ITS2 for *Enicospilus*. Instead *Ophion minutus* was used as the outgroup, since it was recovered as basal by both the COI and 28S datasets, as well as having morphological characters indicating that it is likely basal within *Ophion* (Schwarzfeld et al., in prep.). The final alignment (including partial 28S and 5.8S) was 1307 base pairs in length. However the alignment for the *Ophion scutellaris* group, excluding all other specimens, was 996 base pairs long, with individual sequences ranging from 979–992 base pairs. We successfully sequenced 29 Nearctic and 2 Palearctic specimens within the *Ophion scutellaris* group.

We sequenced the D2-D3 region of 28S rDNA using the following primers: Forward: 5’-GCG AAC AAG TAC CGT GAG GG-3’; Reverse: 5’-TAG TTC ACC ATC TTT CGG GTC-3’ (Laurenne et al. 2006). PCR cycling was 94° for 2 min, 30 cycles of 96° for 15 s, 50° for 30 s, 72° for 30 s and a final extension of 75° for 7 min. Alignment was performed by eye in Mesquite; there were occasional small indels, but generally alignment was unambiguous. The aligned sequences were 725 base pairs in length. We sequenced 8 Nearctic and 1 Palearctic specimen(s) within the *Ophion scutellaris* group, and also included a single sequence of *Ophion scutellaris* from GenBank.

Alignments for all three gene regions have been deposited on TreeBASE, and can be accessed at: http://purl.org/phylo/treebase/phylows/study/TB2:S16341.

### DNA analysis

All molecular analyses were conducted using MEGA version 5 (Tamura et al. 2011). We conducted both maximum likelihood (ML) and maximum parsimony (MP) analyses for the three genes separately. The maximum parsimony analyses were run using heuristic searches with tree-bisection-reconnection, search level 5. We used all sites, 10 starting trees and set max trees to 1000. The best models for the ML analyses were selected in MEGA, using the Bayesian Information Criterion. The following models were selected: COI: TN93+G+I; ITS2: K2+I; 28S: T92+G. The ML search parameters were subtree-pruning-regrafting, with very strong branch swap filter; we used all sites, and the starting tree was obtained using NJ/BioNJ. The trees were tested using both ML and MP bootstrapping in MEGA. The bootstrap analyses used the same parameters as the tree searches, with 1000 replicates for the ML analysis and 10,000 replicates for the MP analysis. We calculated intra- and interspecific sequence divergences using both the Kimura-2-parameter model and as uncorrected p-distances.

### Geometric morphometrics (GM)

We analysed wing morphometrics for 118 specimens (71 female and 47 male; Appendix 1). One sequenced specimen had a missing abdomen, so could not be classified by sex; all other sequenced specimens from the *Ophion scutellaris* group were included in this analysis, as well as in the classical morphometrics analysis below. The right fore and hind wings were removed at the base, soaked briefly in 95% ethanol, and then temporarily slide-mounted in 95% ethanol. Slides were placed on the pane of a lightbox, and photographed using either an 8 megapixel Nikon Coolpix 8400 or a 7.2 megapixel Sony Cybershot DSC-W80 digital camera. The Nikon camera was mounted on a camera mount at a distance of 3.3 cm from the in-focus wing, and wings were photographed using the macro setting and manual focus. The Sony camera was placed at a distance of 4.0 cm from the wing and photographed using the macro setting and autofocus. Several wings were photographed using both cameras to ensure that the two methods were comparable and we concluded that variability between cameras was negligible compared to the variability between specimens. Once photographed, the wings were glued at the base to a small square of cardstock, and included with the specimen as an extra label.

**Figure 1.**
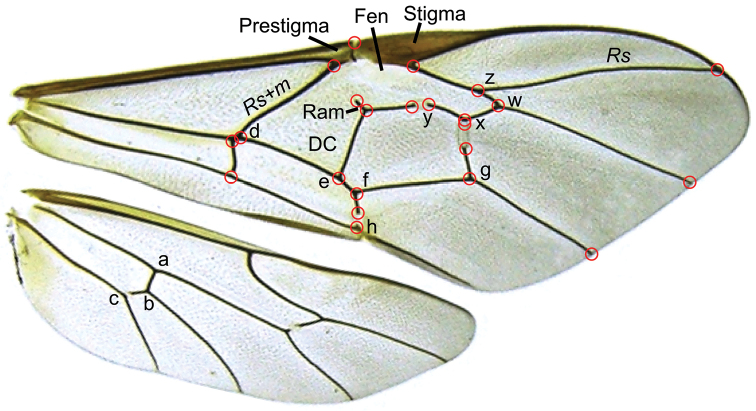
Wing characters used in classical morphometric analysis and in species descriptions. DC = discocubital cell; Fen = fenestra; Ram = ramellus. Wing indices: AI = xy/wz; CI = ef/fh; ICI = wz/wx; SDI = fg/de; cu-a = ab/bc; red circles indicate the locations of the 23 landmarks used for geometric morphometrics.

Only the fore wings were used for the geometric morphometrics. A total of 23 landmarks were digitized in tpsDig version 2.16 (Rohlf 2010) (Figure 1). The landmark data was analyzed in MorphoJ, version 1.03 (Klingenberg 2011). A preliminary analysis showed differences between male and female wings, therefore the sexes were analysed separately. A Procrustes fit was conducted on the male and female datasets to eliminate the variables of position, size and rotation (Rohlf and Slice 1990). We then calculated a covariance matrix of the Procrustes coordinates, and analyzed it using Principal Components Analysis.

### Classical morphometrics (CM)

Eighty-four specimens (52 females and 32 males; Appendix 1) and thirteen morphometric variables were included in this analysis. Eleven of these are ratios of measurements: FW/FM; FW/FH; FW/HW; MS/MW; IOD/OL; OOD/OL; SL/SW; M1B/M1S; M1S/M1A; FL/FW; cu-a (see Table 1 for descriptions). The other two variables are fore wing length and number of flagellomeres. We did not use any fore wing characters (aside from length) so that there is no character overlap between this analysis and the geometric morphometric analysis. The use of ratios, rather than direct measurements, was employed to limit the influence of size on the analysis. However we did include two variables that were not size-corrected (wing length and number of flagellomeres). While it is expected that larger individuals will have more flagellomeres, we have included this variable since there is evidence that the number of flagellomeres can nonetheless be useful for discriminating species of *Ophion*, even among individuals of the same size (Brock 1982). Fore wing length was included since size did seem to be a potential useful discriminating character between species. We conducted Principal Components Analysis of the morphometric data in Gingko version 1.7 (Bouxin 2005), with sexes analyzed separately.

**Table 1.** List of abbreviations for morphological characters included in this study. Abbreviations marked with an asterisk were included in the classical morphometric analysis of the *Ophion scutellaris* species group.

Abbreviation	Character	Details
*Head*		
CAW	Clypeus apical width	A straight line across ventral margin of clypeus (Figure 2a)
CH	Clypeus height	At centre of clypeus, from ventral margin to epistomal sulcus (Figure 2a)
F1	Flagellomere 1 length/width	In ventral view at midpoint of flagellomere
F20	Flagellomere 20 length/width	In ventral view at midpoint of flagellomere
Flag*	Number of flagellomeres	For the morphometric analysis, three specimens of *Ophion idoneus* (one male and two females) had broken antennae. The number of flagellomeres for each of these specimens was estimated by averaging the number of flagellomeres for all remaining *Ophion idoneus* of the respective sex. One female specimen of an unknown species had broken antennae; since the analysis did not allow missing data, the number of flagellomeres was estimated by averaging the number of flagellomeres of all female specimens of a similar size (i.e. excluding *Ophion aureus* and *Ophion keala*).
FH*	Face height	From the ventral margin of the clypeus to the bottom of the facial tubercle in frontal view (Figure 2a)
FM*	Face maximum width	Widest part of the face between the maximum indentation of the eyes (Figure 2a)
FW*	Face width	Between the inner eye margins at the level of the clypeal foveae (Figure 2a)
GI	Genal inflection	Length of the hypostomal carina between base of mandible and intersection with genal carina (Brock 1982)
HW*	Head width	Across the widest part of the eyes in frontal view (Figure 2a)
IOD*	Interocellar distance	Shortest distance between the posterior ocelli in dorsal view
MS*	Malar space	Shortest distance between the eye and the base of the mandible (Figure 2a)
MW*	Mandible width	Measured at the base (Figure 2a)
OC	Postocellar vertex	Minimum distance between occipital carina and posterior ocellus
OL*	Ocellus length	Maximum length of the posterior ocellus
OOD*	Ocellar-ocular distance	Shortest distance from the eye margin to the deepest part of the sulcus adjacent to the posterior ocellus
*Mesosoma*		
SL*	Scutellum length	From the base to the apex of the scutellum
SW*	Scutellum width	Across the base of the scutellum between the inner margins of the lateral scutellar carinae
*Metasoma*		
M1A*	Metasomal segment 1 apical width	The width of the first metasomal segment measured in dorsal view at the apex (Figure 2b)
M1B*	Metasomal segment 1 basal width	The width of the first metasomal segment measured in dorsal view at the base (Figure 2b)
M1S*	Metasomal segment 1 spiracle width	The width of the first metasomal segment measured in dorsal view at the level of the spiracles (Figure 2b)
*Legs*		
CL	Metacoxa length	Maximum length of metacoxa in lateral view
CW	Metacoxa width	Maximum width of metacoxa in lateral view
FL*	Metafemur length	Midpoint of the base of the hind femur to midpoint of the apex, in lateral view
FW*	Metafemur width	Maximum lateral width of the hind femur
MT1	Metatarsomere 1 length	In lateral view at midpoint of tarsomere
MT2	Metatarsomere 2 length	In lateral view at midpoint of tarsomere
MTS	Mesotibial spur ratio	Length of shorter spur/Length of longer spur
*Wings*		
Wing L*	Length of the fore wing	For most specimens, this was measured in ImageJ from the photographs used in the wing geometric morphometric analysis; photographs were calibrated for size using the known size of the coverslip on the slide-mount; for those specimens not photographed for the wing analysis, wing length was measured using a calibrated ocular micrometer
cu-a*	*cu-a* index of hind wing	Length of *cu-a* above *Cu* 1/length of *cu-a* below *Cu* 1 (=ab/bc in Figure 1). For most specimens this was measured in ImageJ 1 46r (Rasband 1997–2012) from the photographs taken for the wing geometric morphometric analysis. For those specimens not photographed (11 specimens), this was measured using an ocular micrometer
AI	Alar index of fore wing	Length of 1*m-cu* between bulla and 2*m-cu*/length of 3*rs-m* (=xy/wz in Figure 1)
CI	Cubital index of fore wing	Length of *Cu* 1 between 1*m-cu* and *Cu* 1a/length of *Cu* 1b (=ef/fh in Figure 1)
ICI	Intercubital index of fore wing	Length of 3*rs-m*/length of *M* between 2*m-cu* and 3*rs-m* (=wz/wx in Figure 1)
SDI	Second discoidal index of fore wing	Length of *Cu* 1a between *Cu* 1b and 2*m-cu*/length of *Cu* 1 between *Rs+M* and 1*m-cu* (=fg/de in Figure 1)

**Figure 2.**
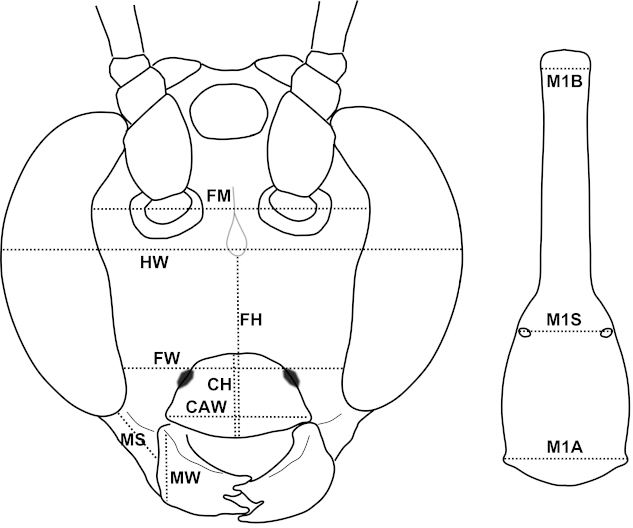
Measurements used in classical morphometric analysis and in species descriptions. **a** Face **b** Metasomal tergite 1, in dorsal view.

## Data resources

The data underpinning the analyses reported in this paper are deposited at Canadensys (www.canadensys.net), and are available at http://dx.doi.org/10.5886/y2bbsq5.

## Results

The *Ophion scutellaris* species group was well-supported by both molecular and morphological characters, and thus far includes seven Nearctic and potentially two Palearctic species (Figure 3a, b, c). The Nearctic species are: *Ophion idoneus* Viereck, 1905, *Ophion clave* Schwarzfeld, sp. n., *Ophion aureus* Schwarzfeld, sp. n., *Ophion brevipunctatus* Schwarzfeld, sp. n., *Ophion dombroskii* Schwarzfeld, sp. n., *Ophion keala* Schwarzfeld, sp. n. and *Ophion importunus* Schwarzfeld, sp. n.. The Palearctic species have been tentatively labeled *Ophion scutellaris* A and *Ophion scutellaris* B. Briefly, the group can be characterized as early-flying, reddish specimens, lacking extensive yellow markings, with the mid-tibial spurs nearly equal in length. The complete diagnosis and description of the species group is provided in the Taxonomic Part below.

### Summary of molecular analyses

Statistics from the molecular analyses are found in Table 2. Maximum likelihood and maximum parsimony analyses gave essentially equivalent results, and there was no conflict between the three genes. Despite the congruence between datasets, we did not include a combined analysis, since the phylogenetic signal from COI was much stronger than in the other two genes. The combined analysis was therefore nearly identical to the COI analysis. Uncorrected p-distances and distances calculated under the K2P model were very similar (Table 3). Throughout the paper we have reported the uncorrected value; if the K2P value differed, we included it in parentheses.

**Table 2.** Summary of statistics from maximum likelihood (ML) and maximum parsimony (MP) analyses of COI, ITS2 and 28S genetic markers.

Analysis	Statistics	COI	ITS2	28S
ML	Log likelihood	-3545.81	-4076.72	-1429.26
MP	Tree Length	579	467	75
	No. MP trees	8	10	10
	CI/RI	0.55/0.80	0.90/0.93	0.88/0.78

**Table 3.** Interspecific and intraspecific percent sequence divergence for three genetic markers within the *Ophion scutellaris* species group. Genetic distances calculated with the Kimura-2-parameter model are found above the diagonal, uncorrected p-distances are below the diagonal, and intraspecific distances are along the diagonal. Intraspecific distances and all 28S distances were identical using both models. *aur* = *aureus*; *bre* = *brevipunctatus*; *cla* = *clave*; *dom* = *dombroskii*; *ido* = *idoneus*; *imp* = *importunus*; *kea* = *keala*; *scu* A = *scutellaris* A; *scu* B = *scutellaris* B.

a. COI									
	*aur*	*bre*	*cla*	*dom*	*ido*	*imp*	*kea*	*scu* A	*scu* B
*aur*	**0.4**	2.0–2.4	2.9–3.7	2.1–2.4	9.5–10.1	9.1–9.6	9.3–10.0	9.0–10.0	8.8–8.9
*bre*	1.9–2.4	**n/a**	3.0–3.4	1.0	8.3–8.5	7.7	8.3–8.7	8.8–9.3	8.5
*cla*	2.8–3.6	3.0–3.3	**0.1–0.3**	3.2–3.5	9.1–9.7	9.0–9.3	9.2–9.8	9.0–9.6	8.7–9.0
*dom*	2.1–2.4	1.0	3.1–3.4	**n/a**	9.5–9.7	8.8	9.2–8.8	9.4–9.8	8.8
*ido*	8.9–9.4	7.9–8.0	8.6–9.0	8.9–9.0	**0–0.3**	2.3–2.4	4.6–5.4	6.5–7.6	6.7–7.0
*imp*	8.6–9.0	7.3	8.4–8.7	8.3	2.2–2.4	**n/a**	4.8–5.3	6.7–7.6	7.0
*kea*	8.7–9.3	7.9–8.2	8.6–9.2	8.6–8.9	4.4–5.2	4.6–5.0	**0.1–0.7**	4.6–5.9	5.4–5.9
*scu* A	8.5–9.2	8.3–8.7	8.5–9.0	8.8–9.1	6.2–7.1	6.4–7.1	4.5–5.6	**0**	3.4–3.8
*scu* B	8.3–8.4	8.0	8.1–8.4	8.3	6.4–6.7	6.7	5.2–5.6	3.3–3.6	**n/a**

b. ITS2								
	*aur*	*bre*	*cla*	*dom*	*ido*	*imp*	*kea*	*scu*
*aur*	**0**	0.6	0.2–0.3	0.3	2.0–2.1	2.3–2.4	1.9–2.0	1.8–1.9
*bre*	0.6	**n/a**	0.5	0.3	2.3	2.6	2.2	2.1
*cla*	0.2–0.3	0.5	**0**	0.1	1.8	2.1	1.7	1.6–1.7
*dom*	0.3	0.3	0.1	**n/a**	1.9	2.2	1.8	1.7
*ido*	1.9–2.1	2.2–2.3	1.7–1.8	1.8	**0**	0.9	0.1	0.4–0.5
*imp*	2.3–2.4	2.6	2.0–2.1	2.1	0.9	**n/a**	0.8	0.7–0.8
*kea*	1.8–1.9	2.1	1.6	1.7	0.1	0.8	**0**	0.3–0.4
*scu*	1.7–1.9	2.0–2.1	1.5–1.7	1.6–1.7	0.4–0.5	0.7–0.8	0.3–0.4	**0.1**

c. 28S; p-distances and K2P distances were identical, and are therefore only reported above the diagonal								
	*aur*	*bre*	*cla*	*dom*	*ido*	*imp*	*kea*	*scu*
*aur*	**n/a**	0.1	0.1	0.1	0.6	0.7	0.6	0.6–0.7
*bre*	0.1	**n/a**	0	0	0.4	0.6	0.4	0.5–0.6
*cla*			**n/a**	0	0.4	0.6	0.4	0.5–0.6
*dom*				**n/a**	0.4	0.6	0.4	0.5–0.6
*ido*					**n/a**	0.1	0	0–0.1
*imp*						**n/a**	0.1	0.2–0.3
*kea*							**0**	0–0.1
*scu*								**n/a**

Monophyly of the *Ophion scutellaris* species group was strongly supported in almost all analyses, with bootstrap support ranging from 72% in the 28S ML analysis to 100% in the ITS2 MP analysis (Figure 3a, b, c). Only the MP analysis of the 28S data lacked bootstrap support for the species group. Within the species group there was a further strongly supported division into two subgroups (A and B, Figure 3a, b, c); these two subgroups were recovered by all genes and all analyses, with bootstrap support ranging from 72% (28S MP) to 100% (COI ML). The Palearctic species, *Ophion scutellaris*, was recovered within subgroup A. The five specimens identified as this species formed a well-supported clade in the COI analysis (ML bootstrap: 99, MP: 95). However there was also a divergence of 3.6% (K2P: 3.8%) between one specimen (*Ophion scutellaris* B) and the remaining *Ophion scutellaris* specimens, which may indicate a previously unrecognized species. Only one of these putative species was sequenced for ITS2.

**Figure 3a.**
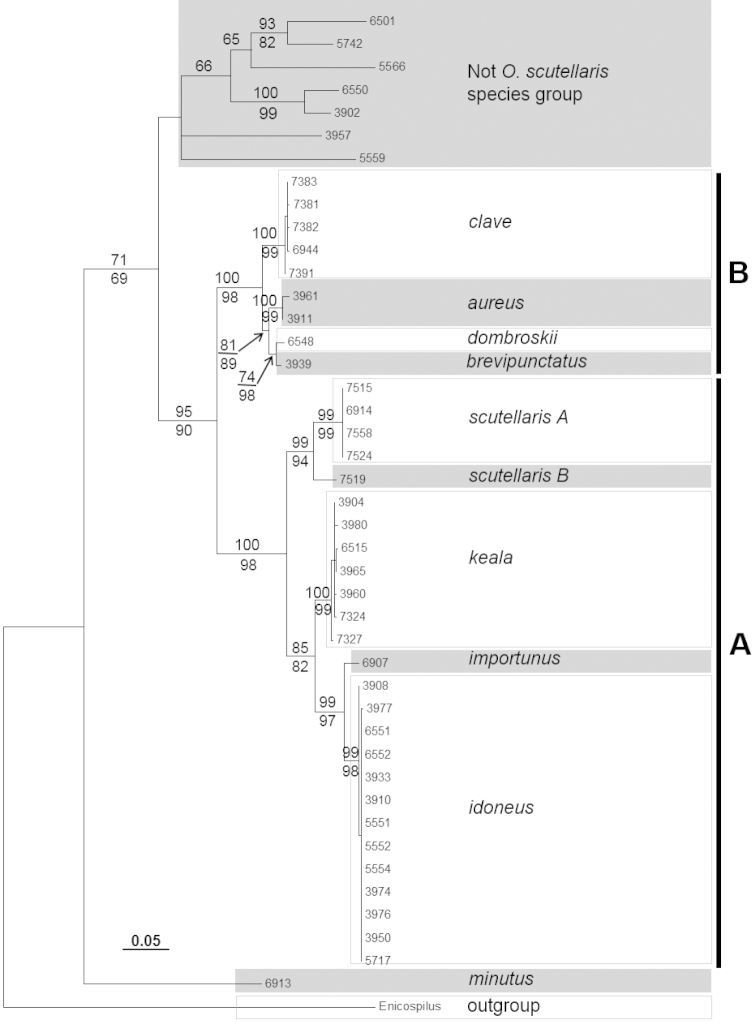
Maximum likelihood tree of COI sequences. Maximum likelihood bootstrap support values are above branches and maximum parsimony bootstrap values are below branches.

**Figure 3b.**
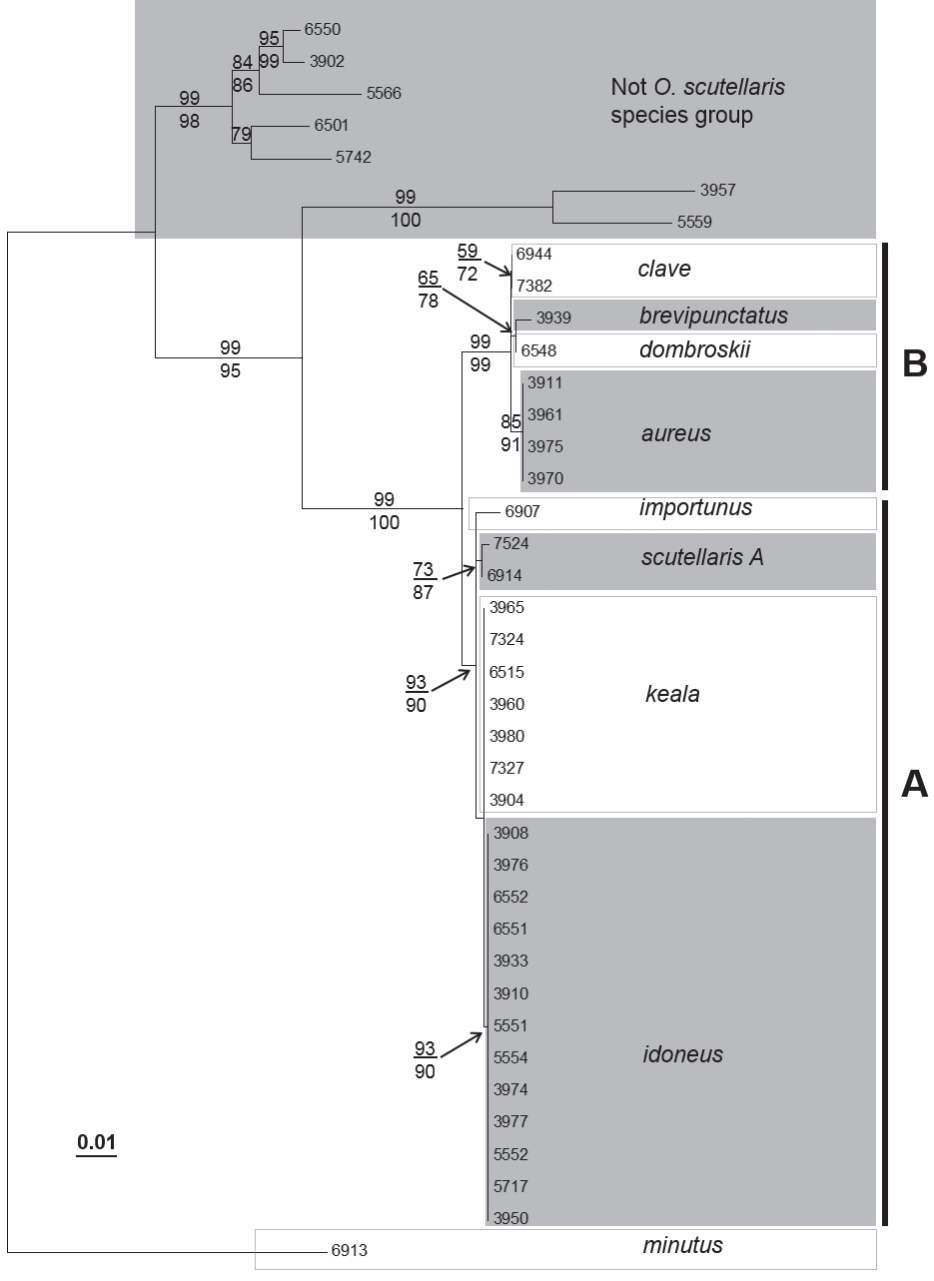
Maximum likelihood tree of ITS2 sequences. Maximum likelihood bootstrap support values are above branches and maximum parsimony bootstrap values are below branches.

**Figure 3c.**
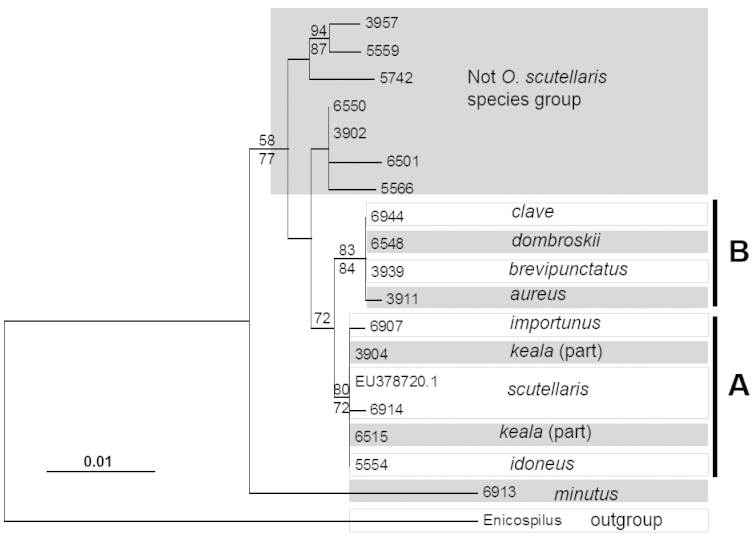
Maximum likelihood tree of 28S sequences. Maximum likelihood bootstrap support values are above branches and maximum parsimony bootstrap values are below branches.

Sequence divergence within the *Ophion scutellaris* group was highest in the COI dataset, with a maximum of 9.4% (K2P: 10.1%) sequence divergence within the species group, and 14.8% (K2P: 16.6%) within all included *Ophion*. While ITS2 had a higher sequence divergence overall (17.4%; K2P: 19.9%), the divergence within the *Ophion scutellaris* species group was only 2.6%. 28S was highly conserved, with a 2.8% (K2P: 2.9%) divergence within all *Ophion*, and 0.7% within the *Ophion scutellaris* group.

### Summary of morphometric analyses

**Figure 4.**
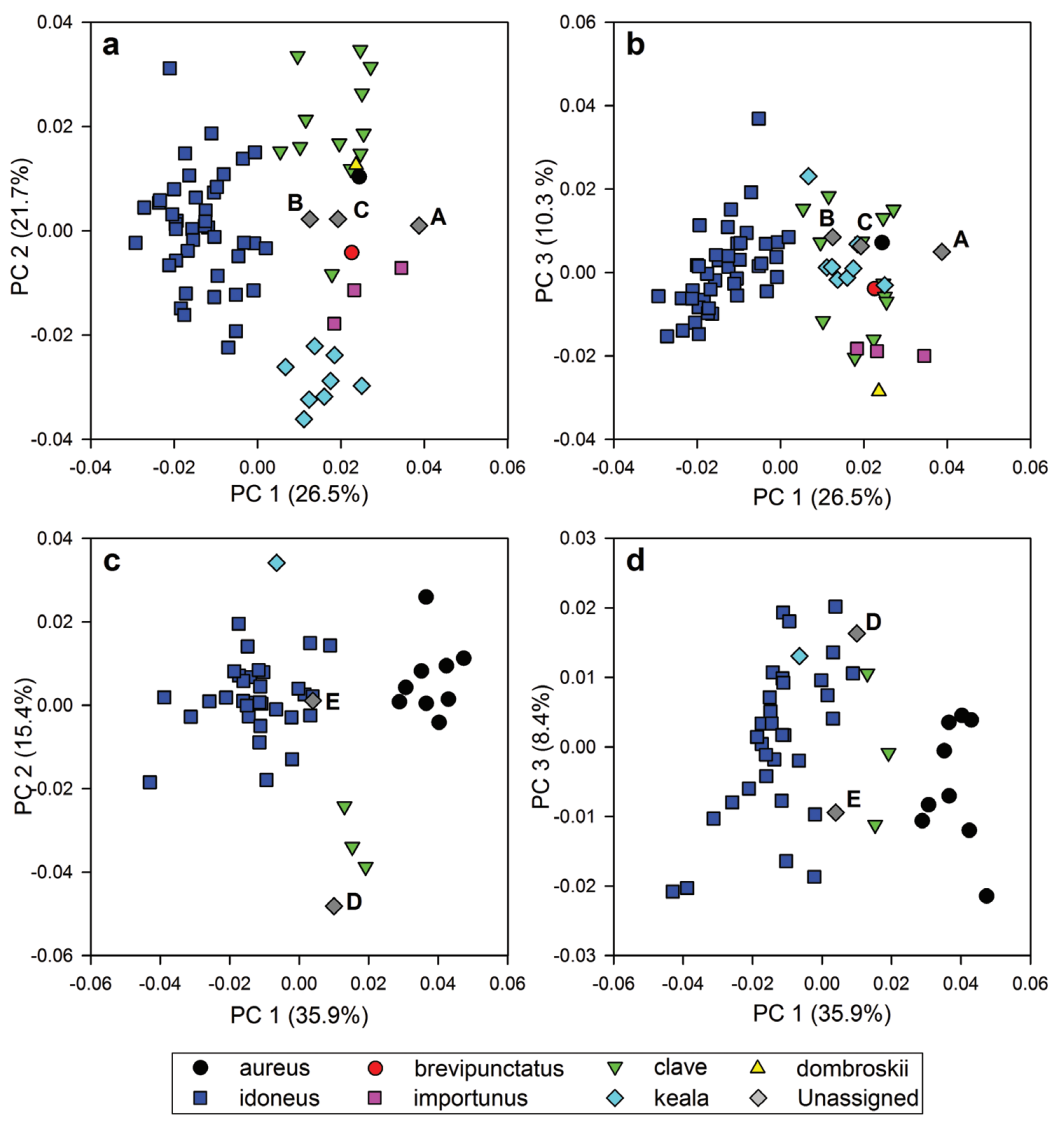
Principal Components Analysis of 23 fore wing landmarks from female (**a, b**) and male (**c, d**) specimens of the *Ophion scutellaris* species-group. Upper-case letters (**A–E**) refer to unassigned specimens (see text).

For the geometric morphometric (GM) analysis, approximately 60% of the variation in wing shape could be summarized in the first three principal components (PC). In the analysis of females, the variation was quite evenly represented by the first two principal components (PC1: 26.5%, PC2: 21.7%, PC3: 10.3%; Figure 4a, b), while in the analysis of males, most of the variation was explained in the first PC (PC1: 35.9%, PC2: 15.4%, PC3: 8.4%; Figure 4c, d).

**Figure 5.**
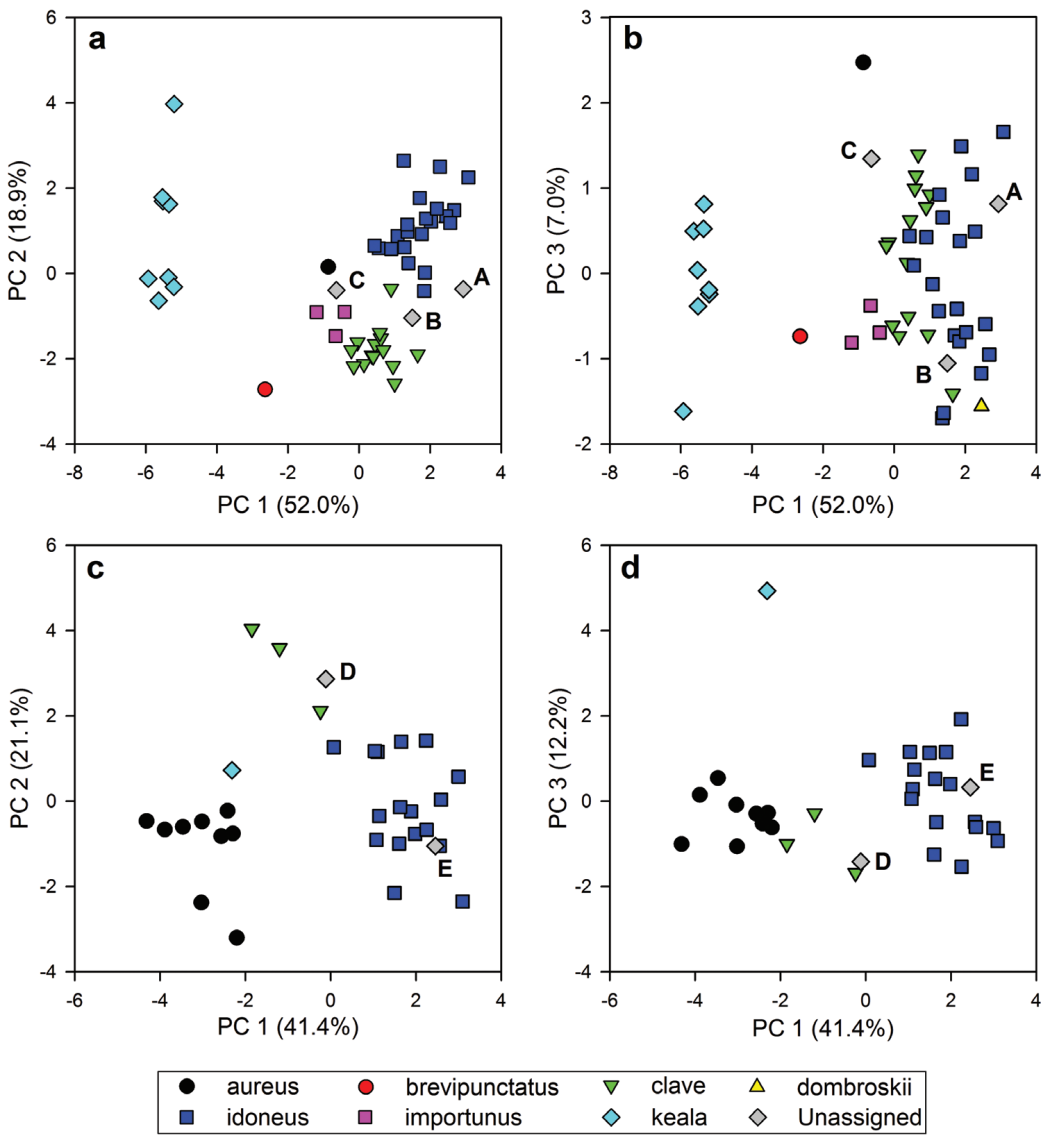
Principal Components Analysis of 13 morphometric characters for female (**a, b**) and male (**c, d**) specimens of the *Ophion scutellaris* species-group. Upper-case letters (**A–E**) refer to unassigned specimens (see text).

In the classical morphometric (CM) analysis, the first three principal components represented 77.9% of the total variation in the female dataset (PC1: 52.0%, PC2: 18.9%, PC3: 7.0%; Figure 5a, b). For the analysis of males, they represented 74.8% of the variation (PC1: 41.4%, PC2: 21.1%, PC3: 12.2%; Figure 5c, d). All measurements are summarized in Tables 4 and 5.

**Table 4.** Summary of measurements of 13 morphometric variables for females of the *Ophion scutellaris* species group. Morphometric variables are defined in Table 1. The mean ± standard deviation for each variable is given, followed by the range. *cla* = *clave*; *aur* = *aureus*; *bre* = *brevipunctatus*; *dom* = *dombroskii*; *kea* = *keala*; *imp* = *importunus*; *ido* = *idoneus*; A, B, C refer to unplaced specimens; the number of specimens measured for each species is in parentheses.

Variable	*cla* (13)	*aur* (1)	*bre* (1)	*dom* (1)	*kea* (8)	*imp* (3)	*ido* (21)	A (1)	B (1)	C (1)
FW/FM	0.83±0.02	0.83	0.79	0.84	0.81±0.02	0.82±0.01	0.86±0.01	0.88	0.87	0.83
	*0.81–0.85*				*0.78–0.84*	*0.82–0.83*	*0.84–0.89*			
FW/FH	1.23±0.08	1.37	1.27	1.34	1.33±0.09	1.26±0.02	1.37±0.05	1.30	1.29	1.29
	*1.19–1.26*				*1.24–1.49*	*1.25–1.29*	*1.28*–*1.45*			
FW/HW	0.52±0.02	0.51	0.47	0.53	0.48±0.01	0.50±0.00	0.53±0.01	0.54	0.54	0.51
	*0.50–0.54*				*0.46–0.50*	*0.49–0.50*	*0.51–0.55*			
MS/MW	0.51±0.10	0.60	0.50	0.58	0.40±0.04	0.51±0.06	0.59±0.04	0.60	0.58	0.54
	*0.43–0.63*				*0.33–0.47*	*0.46–0.58*	*0.54–0.70*			
IOD/OL	0.77±0.08	0.71	0.69	1.20	0.36±0.06	0.72±0.16	0.74±0.08	1.00	0.73	0.71
	*0.53–1.00*				*0.27–0.44*	*0.53–0.85*	*0.61–0.86*			
OOD/OL	0.22±0.05	0.29	0.13	0.45	0.16±0.04	0.19±0.04	0.33±0.04	0.32	0.20	0.21
	*0.13–0.29*				*0.11–0.22*	*0.15–0.23*	*0.25–0.43*			
SL/SW	1.43±0.17	1.18	1.48	1.76	1.63±0.10	1.58±0.03	1.52±0.09	1.39	1.52	1.45
	*1.23–1.58*				*1.47–1.76*	*1.55–1.60*	*1.37–1.69*			
M1B/M1S	0.80±0.11	0.70	0.80	0.78	0.63±0.06	0.78±0.04	0.80±0.06	0.82	0.88	0.63
	*0.71–0.92*				*0.57–0.74*	*0.75–0.82*	*0.71–0.92*			
M1S/M1A	0.61±0.07	0.69	0.60	0.64	0.76±0.06	0.66±0.02	0.67±0.06	0.64	0.53	0.70
	*0.58–0.67*				*0.57–0.74*	*0.64–0.68*	*0.59–0.79*			
FL/FW	7.82±0.95	7.95	9.50	7.24	10.26±1.09	8.58±0.31	6.87±0.26	7.56	7.89	7.79
	*7.00–8.59*				*8.41–11.63*	*8.25–8.87*	*6.41–7.50*			
Flag	55.08±5.82	65	67	51	70.63±1.30	55.00±1.73	54.05±1.93	52	59	59
	*52–60*				*69–73*	*54–57*	*51–57*			
cu-a	0.69±0.11	0.88	0.66	0.83	1.08±0.13	0.72±0.09	0.83±0.12	0.62	0.68	0.64
	*0.60–0.84*				*0.89–1.26*	*0.66–0.83*	*0.68–1.13*			
Wing L	11.83±2.76	16.69	14.04	10.80	19.06±0.45	12.92±0.38	10.13±0.64	10.70	12.86	12.70
	*10.49–12.44*				*18.64–19.73*	*12.57–13.33*	*8.98–11.48*			

**Table 5.** Summary of measurements of 13 morphometric variables for males of the *Ophion scutellaris* species group. Morphometric variables are defined in Table 1. The mean ± standard deviation for each variable is given, followed by the range. *cla* = *clave*; *aur* = *aureus*; *kea* = *keala*; *ido* = *idoneus*; D, E refer to unplaced specimens; the number of specimens measured for each species is in parentheses.

Variable	*cla* (3)	*aur* (9)	*kea* (1)	*ido* (17)	D (1)	E (1)
FW/FM	0.85±0.02	0.84±0.01	0.86	0.89±0.02	0.83	0.91
	*0.83–0.87*	*0.82–0.89*		*0.85–0.91*		
FW/FH	1.22±0.04	1.33±0.05	1.41	1.40±0.03	1.23	1.39
	*1.18–1.26*	*1.25–1.41*		*1.32–1.45*		
FW/HW	0.50±0.02	0.52±0.01	0.50	0.53±0.01	0.50	0.55
	*0.49–0.52*	*0.50–0.54*		*0.51–0.56*		
MS/MW	0.48±0.06	0.74±0.05	0.39	0.65±0.08	0.58	0.70
	*0.43–0.54*	*0.65–0.80*		*0.50–0.82*		
IOD/OL	0.79±0.13	0.74±0.05	0.44	0.87±0.16	1.04	0.90
	*0.67–0.92*	*0.65–0.80*		*0.58–1.25*		
OOD/OL	0.23±0.03	0.30±0.02	0.44	0.48±0.14	0.33	0.40
	*0.20–0.27*	*0.26–0.33*		*0.35–0.92*		
SL/SW	1.54±0.12	1.23±0.08	1.74	1.44±0.12	1.68	1.44
	*1.43–1.67*	*1.13–1.38*		*1.21–1.68*		
M1B/M1S	0.79±0.04	0.64±0.08	0.63	0.77±0.07	0.79	0.75
	*0.76–0.84*	*0.54–0.78*		*0.61–0.87*		
M1S/M1A	0.65±0.06	0.72±0.04	0.79	0.73±0.07	0.67	0.80
	*0.61–0.71*	*0.67–0.78*		*0.62–0.90*		
FL/FW	7.08±0.09	8.72±0.54	9.03	6.95±0.43	7.13	7.14
	*7.00–7.18*	*8.11–9.63*		*6.31–7.85*		
Flag	54.67±1.53	66.78±1.56	65	54.69±1.74	54	52
	*53–56*	*65–69*		*51–57*		
cu-a	0.69±0.09	0.69±0.10	1.43	0.97±0.22	0.76	0.96
	*0.59–0.74*	*0.51–0.83*		*0.72–1.53*		
Wing L	10.78±0.11	15.55±0.94	16.23	9.45±0.56	9.91	8.91
	*10.65–10.85*	*14.37–17.07*		*8.42–10.74*		

### Morphological characterization of subgroups

**Figure 6.**
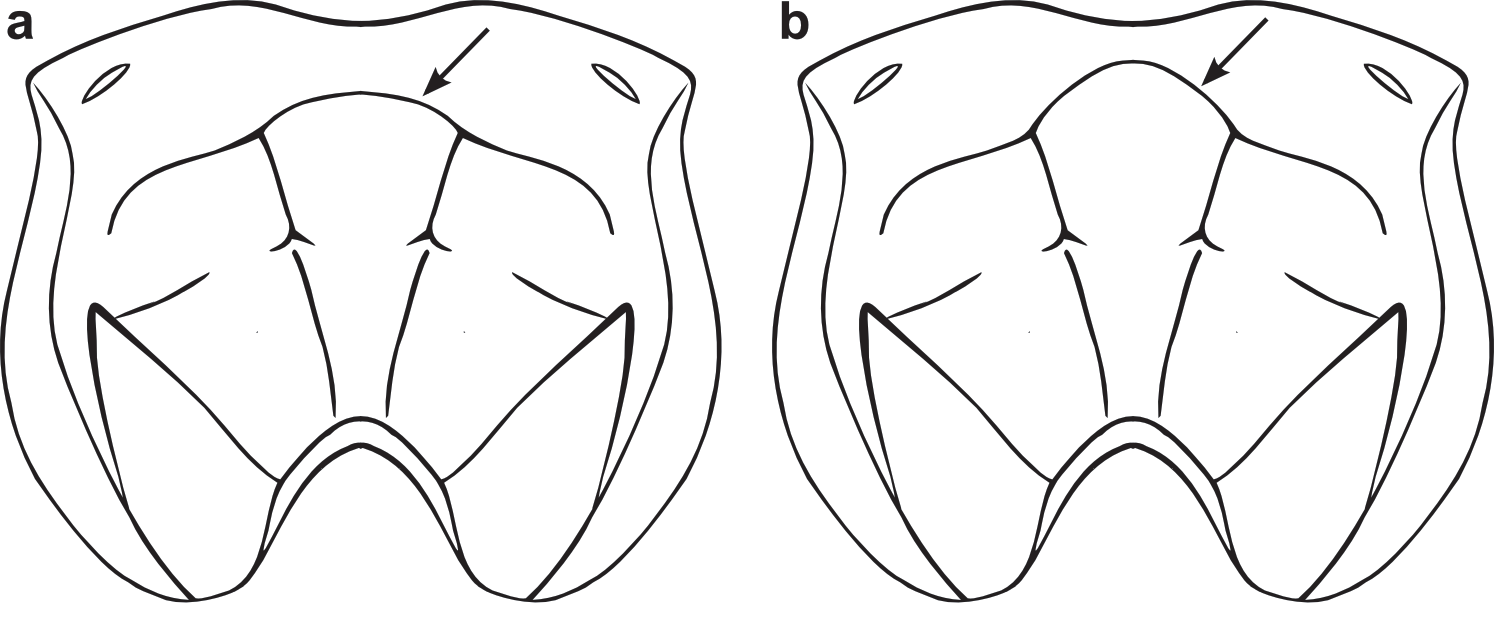
Generalized propodeum demonstrating the difference in the anterior transverse carina (ATC) between the two subgroups of the *Ophion scutellaris* species group. **a** Subgroup A, showing the weakly arched ATC **b** Subgroup B, showing the strongly arched ATC.

**Figure 7.**
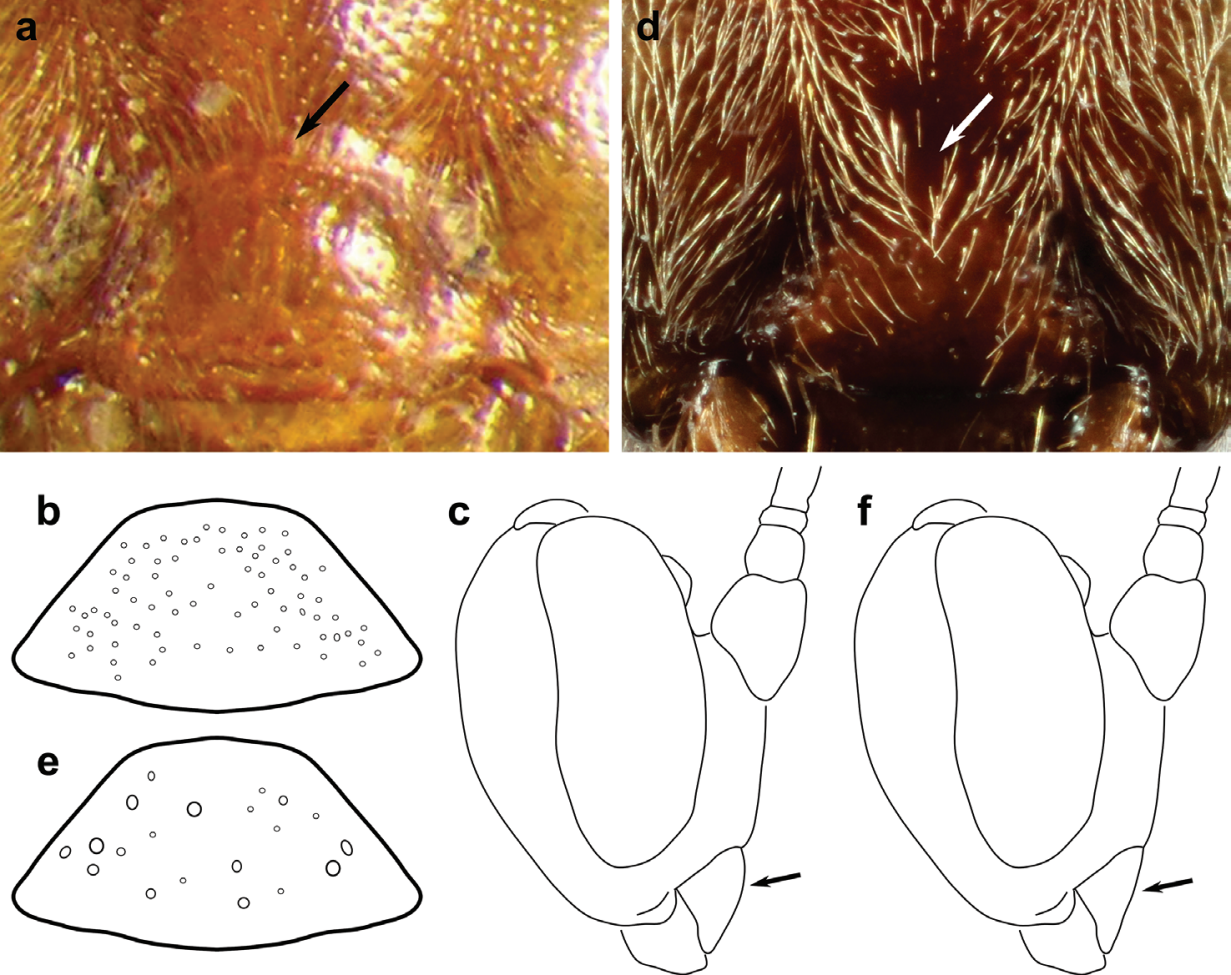
Clypeus of subgroup A (**a–c**) and subgroup B (**d–f**) within the *Ophion scutellaris* species group. **a, d** Clypeus in frontal view; arrow indicates epistomal sulcus **b, e** Clypeus in frontal view, showing representative clypeal punctures **c, f** Clypeus in lateral view; arrow indicates the more convex clypeus in subgroup A, compared to subgroup B.

Examination of specimens from the two strongly supported subgroups from the molecular analysis uncovered additional morphological characters distinguishing them. The most useful character to distinguish these subgroups can be found on the propodeum. All specimens in subgroup A have a weakly arched anterior transverse carina (Figure 6a), while subgroup B has this carina strongly arched centrally (Figure 6b). In addition, the clypeus of species in subgroup A tends to be more convex and more strongly separated from the face (i.e. with a more deeply impressed epistomal sulcus), with small regular punctures (Figure 7a–c). In comparison, species in subgroup B have a flatter clypeus that is more weakly separated from the face, with larger, irregularly spaced punctures (Figure 7d–f).

### Species discovery and delimitation

Six Nearctic species are described, and one redescribed, based on the integrated results of these analyses (see Taxonomic Part below). Because of the nature of the iterative process, a single path was not followed in determining the species boundaries for all species.

**Plate 1.**
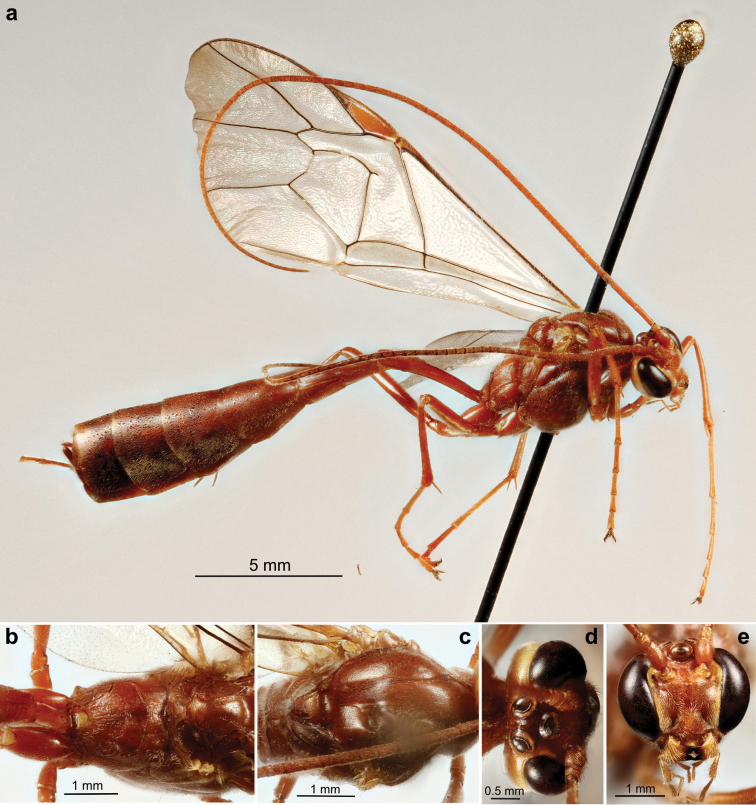
*Ophion keala* Schwarzfeld sp.n., type specimen; **a** habitus **b** propodeum and scutellum **c** mesothorax **d** head, dorsal **e** face.

Two species (*Ophion aureus* Schwarzfeld, sp. n. and *Ophion keala* Schwarzfeld, sp. n.) were easily recognizable as distinct based on gross morphology. In particular, they are both considerably larger than the remaining species in the group. *Ophion aureus* is only known from the Peace River region of Alberta, and was recognized based on its golden-orange colour and distinctive propodeal carinae (Plate 5). It formed a strongly supported clade in both the COI and ITS2 analyses (Figure 3a, b, c). The minimum interspecific divergence was 1.9% (K2P: 2.0%) divergence for COI, and 0.2% for ITS2 (Table 3a). There was an intraspecific divergence of 0.4% for the two specimens that were successfully sequenced for COI, and the four specimens that were sequenced for ITS2 were invariant. *Ophion keala* is the largest species in this group (as well as being among the largest species of *Ophion* we have examined) and is a uniformly dark red colour (Plate 1). In the analysis of COI, it formed a strongly supported monophyletic clade, and was separated by a minimum of 4.4% sequence divergence (K2P: 4.6%) from any other species. However, according to ITS2, it was recovered as a paraphyletic grade with respect to another species, *Ophion idoneus*, from which it differed by 0.1% sequence divergence. The molecular analyses recovered *Ophion aureus* within subgroup B and *Ophion keala* within subgroup A, and the morphology of each is consistent with the respective subgroups.

There is a strong sex bias in the specimens available for each of these species. *Ophion aureus* was represented entirely by males, except for a single female specimen, while the opposite occurred in *Ophion keala*. They could therefore only be thoroughly assessed in the morphometric analyses of male or female specimens, respectively. Both species were successfully delimited by both GM and CM analyses, with essentially no overlap with other specimens (Figures 4, 5). Only the plot of PC1 vs PC3 in the GM analysis failed to recognized *Ophion keala* as distinct. The singleton specimens of the opposite sex were also included in the respective analyses. While PCA maximizes the spread of variation of all specimens, and therefore is less informative for singletons, these single specimens were also often recovered as distinct from all other clusters, particularly in the CM analysis.

**Plate 2.**
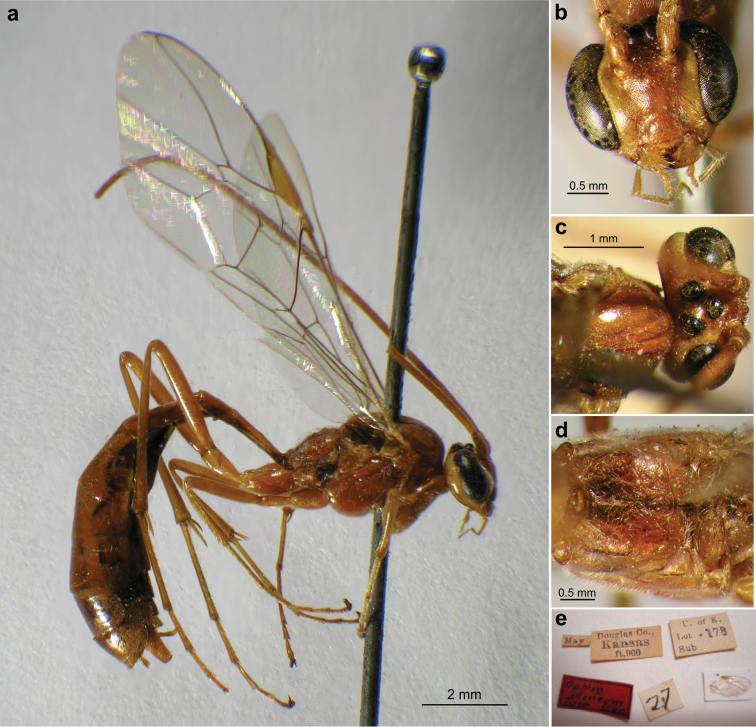
*Ophion idoneus* Viereck, type specimen; **a** habitus **b** face **c** head, dorsal **d** propodeum **e** labels.

*Ophion idoneus* Viereck, the only previously described Nearctic species within this species group, was also initially recognized on the basis of morphology, in particular the shape of the propodeal carinae, the lack of a ramellus in the fore wing and the small size (Plate 2). It was identified to species by comparison to the type specimen. The molecular analyses recovered it within subgroup A, and it was strongly supported by both COI and ITS2 (Figure 3a, b, c). The minimum interspecific difference was 2.2% (K2P: 2.3%) for COI, and 0.1% for ITS2. It was almost invariant intraspecifically, with a maximum divergence of 0.3% according to COI (equivalent to two base pairs), while all ITS2 sequences were identical. It was also recovered as distinct in all morphometric analyses, with only the plot of PC1 vs PC3 in the CM study having significant overlap with any other group (Figures 4, 5). It was by far the most common species in this species group.

**Plate 3.**
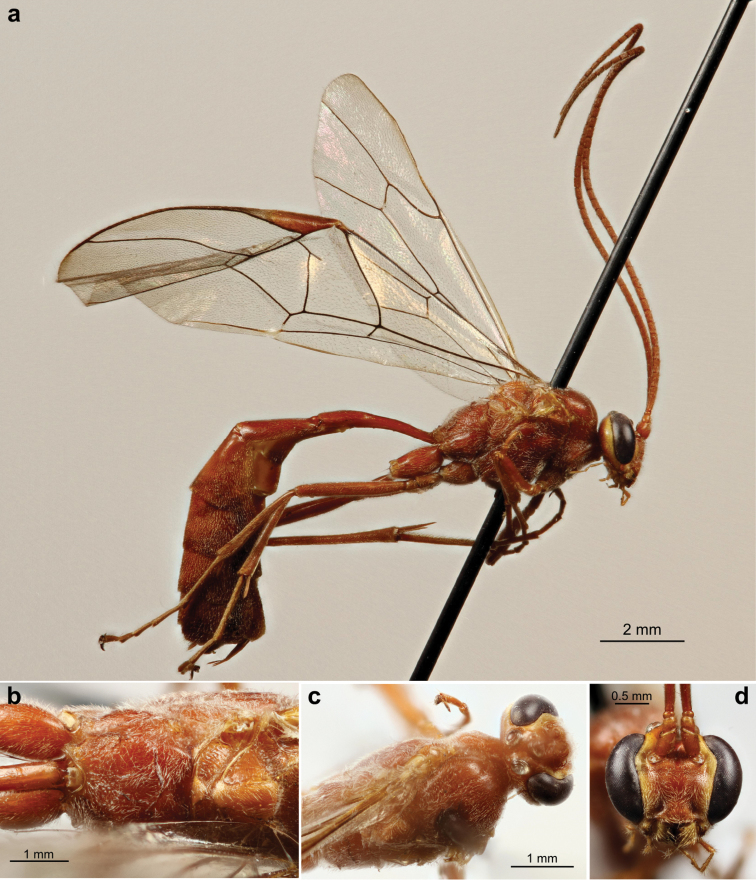
*Ophion importunus* Schwarzfeld sp.n., type specimen; **a** habitus **b** propodeum and scutellum **c** mesothorax and head **d** face.

A single female specimen of *Ophion importunus* sp. n. was first noted as being very similar to *Ophion idoneus*, but differing slightly in the shape of the propodeal carinae, along with being slightly larger and having a longer ramellus (Plate 3, Figure 11c). Sequencing of this specimen confirmed it as being related to *Ophion idoneus*, with all analyses strongly supporting its inclusion within subgroup A. However it was separated from *Ophion idoneus* by a minimum of 2.2% (K2P: 2.3%) divergence according to COI and 0.9% divergence according to ITS2, compared to the almost invariant sequences within *Ophion idoneus* (Table 3). Two other female specimens were identified morphologically as belonging to subgroup A, and were hypothesized to be conspecific with *Ophion importunus*. Both GM and CM analyses supported this hypothesis, with the three specimens grouping closely together, while being distinct from the cluster of *Ophion idoneus* (Figures 4a, b; 5a, b).

*Ophion clave* Schwarzfeld, sp. n. was the only species other than *Ophion idoneus* that was represented by multiple male and female specimens, though female specimens were more common. It was first identified as a putative species based on the molecular analyses. It was recovered as part of subgroup B by all analyses, and formed a well-supported monophyletic clade in the analysis of COI (Figure 3a); only two specimens were successfully sequenced for ITS2, however these were also recovered as a clade, with moderate bootstrap support (Figure 3b). It had a minimum interspecific divergence of 2.8% (K2P: 2%) for COI, and a maximum intraspecific divergence of 0.3%. For ITS2, there was a minimum interspecific divergence of 0.1% (a single base pair), while the two sequences within this species were invariant. Based on these results, the specimens were examined for morphological characters and a number of additional, unsequenced specimens were hypothesized to be part of this species.

The morphometric analyses generally recovered both males and females of this species as distinct clusters compared to other species. For the analysis of females, this result was strongest in the plots of PC1 vs PC2 of both GM and CM, whereas all plots from the analysis of males recovered this species as a distinct group (Figures 4, 5). In a few cases, female specimens that were originally unassigned to any species also clustered with this group in both GM and CM analyses. Based on a qualitative examination of these specimens, we further refined our morphological species concept for this species. In the GM analysis, a single female specimen was not recovered with the rest of the species. However, morphological examination of this specimen supports its assignment to *Ophion clave*, as does the CM analysis. This indicates that wing shape outliers can exist within species, though misidentification cannot be conclusively ruled out.

**Plate 4.**
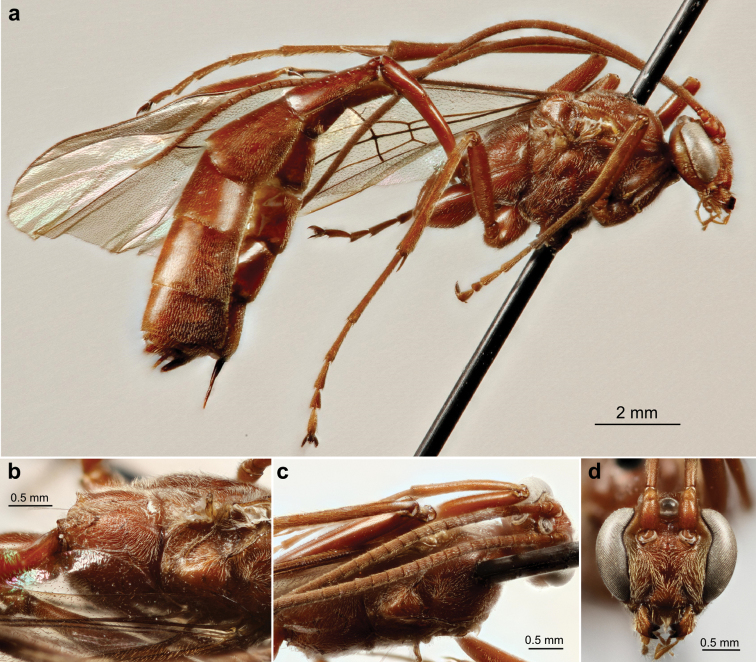
*Ophion clave* Schwarzfeld sp.n., type specimen; **a** habitus **b** propodeum and scutellum **c** mesothorax and head **d** face.

*Ophion brevipunctatus* Schwarzfeld, sp. n. and *Ophion dombroskii* Schwarzfeld, sp. n. are each represented by singletons in this study. *Ophion dombroskii* differs from all other members of the *scutellaris* species group, and from all other known Nearctic *Ophion*, by its distinctive black face and thorax (Plate 7). In comparison, *Ophion brevipunctatus* looks superficially very similar to *Ophion clave* (Plates 6, 4). However both ITS2 and COI recovered these two specimens as a monophyletic clade, with bootstrap support ranging from 65% (ITS2 ML) to 98% (COI MP). They were separated by only 1.0% sequence divergence according to COI, and by 0.3% divergence in ITS2 (Table 3). Neither morphometric analysis, however, showed any similarity between these two specimens, which supports the view that they are not merely colour morphs of the same species. Qualitatively, *Ophion dombroskii* has unusually short antennal segments, which, along with the black colouration, suggest this species is active diurnally (Gauld 1980). While PCA will not necessarily distinguish singletons, *Ophion brevipunctatus* was nevertheless recovered as distinct from all other clusters in the CM study, thus supporting the distinctiveness of this species. Further morphological examination uncovered additional characters. In particular, this species can be distinguished from the apparently more common *Ophion clave* by the larger size, extremely shallow and sparse facial punctures, and the lack of any yellow on the orbits (Plate 6).

Five specimens (3 female and 2 male) remain unassigned to any species. Based on the structure of the propodeal carinae, one of the female specimens can be assigned to subgroup A, while the other two are part of subgroup B. The former specimen (labeled as “A” in figures and tables) was initially identified as *Ophion idoneus*, but based on its divergent position in the morphometric analyses, particularly in the GM analysis, we re-examined this specimen, and concluded that it also differs from *Ophion idoneus* morphologically. The other two female specimens (“B” and “C”) cluster near or within *Ophion clave* in both CM and GM analyses. However they were sufficiently morphologically divergent from *Ophion clave* and from each other that we suspect this does not indicate conspecificity.

One of the two unplaced male specimens (“D”) can be assigned to subgroup B, and the other (“E”) is in subgroup A. According to both the GM and CM analyses, these clustered with species from their respective subgroup, i.e. “D” was near *Ophion clave* and “E” was near *Ophion idoneus*. However, they are sufficiently different morphologically from these species that this is probably an artifact of the data. It is possible that specimen “E” represents the unknown male of *Ophion importunus*, whereas we are almost certain that specimen “D” is an additional undescribed species.

## Discussion

The genus *Ophion* is often considered a particularly challenging taxonomic puzzle, due to high intraspecific and low interspecific morphological variation (Townes 1971, Brock 1982). We used an iterative analysis of multiple genetic markers, geometric morphometrics, classical morphometrics and morphology to define the *Ophion scutellaris* species group, and to assess and delimit species within the group. All analyses were broadly congruent, however each provided unique information to aid in understanding this group.

While 28S was useful in supporting the monophyly of the species group as a whole, and particularly of the two subgroups, it was too conserved to distinguish species within each subgroup. COI and ITS2 were both apparently effective at separating species, since most species, as defined through the integrated analysis, were recovered as well supported clades, with greater divergence between species than within species. However, few individuals over a limited geographical range were sequenced for the majority of species; further sequencing is needed to fully assess the ability of these markers to distinguish species across their range (Ekrem et al. 2007, Bergsten et al. 2012). Nevertheless, at least in the case of *Ophion idoneus* the haplotypes were almost invariant across Canada.

Despite the apparent utility of these molecular markers, morphology is an essential component of diagnosing species, since clusters of haplotypes are not necessarily equivalent to biological species (Sperling and Roe 2009, Dupuis et al. 2012). As well, DNA cannot be sequenced for all specimens, and without morphology these species would remain largely unknown (Schlick-Steiner et al. 2007). Finally at least two species in this study, *Ophion brevipunctatus* and *Ophion dombroskii*, would probably be considered a single species based on COI alone, yet were clearly distinct morphologically.

Qualitative morphology, geometric morphometrics and classical morphometrics provide three additional semi-independent datasets with which to evaluate species diagnoses. Each species in this study is distinguishable morphologically, though again small sample sizes and limited geographical sampling mean the morphological variability within each species has probably not been fully sampled. Four species (*Ophion clave*, *Ophion brevipunctatus*, *Ophion importunus*, and *Ophion idoneus*) look superficially very similar, however each has qualitative morphological characters that are sufficient to separate them. In comparison, *Ophion aureus*, *Ophion keala*, and *Ophion dombroskii* are very morphologically distinctive species. Both *Ophion brevipunctatus* and *Ophion dombroskii* are described from single specimens. While some taxonomists argue that species should never be described from singletons (e.g. Dayrat 2005), Lim et al. (2012) counter that rarity is a fact of biodiversity, and that we will consistently underestimate diversity if we ignore singleton species. Finally, while beyond the scope of this study, initial examination of the two putative species of “*Ophion scutellaris*” supports their distinctiveness, with “*scutellaris* B” being larger and having coarser, denser facial punctures.

Principal Components Analysis is a one-group method for data exploration, meaning it does not include *a priori* group designations (Strauss 2010). As such, it will not definitely separate groups, even if they are distinct. Discriminant analysis is more effective for separating predefined clusters of species and assigning specimens to groups, but it requires known species, which have been identified based on other criteria (e.g. hosts, pheromones, genetic markers) (Marcus 1990, Mutanen and Pretorius 2007, Strauss 2010). In this case, sequenced specimens would be ideal, but sample sizes were too small for the analysis to be statistically valid. Basing the analysis on specimens that were identified morphologically would be circular, considering that some specimens were re-examined and re-classified based on the morphometric analyses. We have therefore restricted the analysis to exploratory PCA’s. Future studies are needed to increase molecular sampling over a wider geographic range, which could then be used for discriminant analyses of morphometric data.

Both geometric and classical morphometrics were effective for clustering species, although the clusters were often not widely separated. In general, superficially similar species clustered more closely together than they did to morphologically divergent species, indicating congruence between quantitative measures of shape and subjective, qualitative analysis. Specimens that did not cluster with other members of their putative species were flagged for further examination. In some cases these were misidentifications, while in others morphology and the alternate morphometric analysis supported their placement within a given species. Conversely, inclusion within a cluster did not guarantee that the specimen was a member of that species. These examples further support the advantage of including multiple lines of evidence for accurate species delimitation.

## Conclusion

This is a first attempt at describing species within this newly-defined species group; as such, it should be considered a work in progress. This study was almost entirely limited to Canadian material, most species were from only a few localities, and all species except *Ophion idoneus* were represented by small numbers of individuals. As well, there are almost certainly additional undescribed species in the material available. All of this indicates that we have just begun to sample the true diversity within this species group. Nonetheless, we have shown that by using an iterative analysis of morphology, molecular analysis and morphometrics, we can delimit and describe species within a genus that is so morphologically challenging that no new species have been described in North America for more than a hundred years. Furthermore, molecular and morphological recognition of this species group will now allow more targeted specimen collection and museum research, supporting a global revision of the species group in its entirety.

## Taxonomic part

### Terminology

Morphological terms were matched as closely as possible to the Hymenoptera Anatomy Ontology (Yoder et al. 2010). Further information and images for the majority of terms can thus be found at http://portal.hymao.org. Some additional terms were included from the ophionine literature, primarily Brock (1982) and Gauld (1991); these terms are defined below and in Table 1.

**Figure 8.**
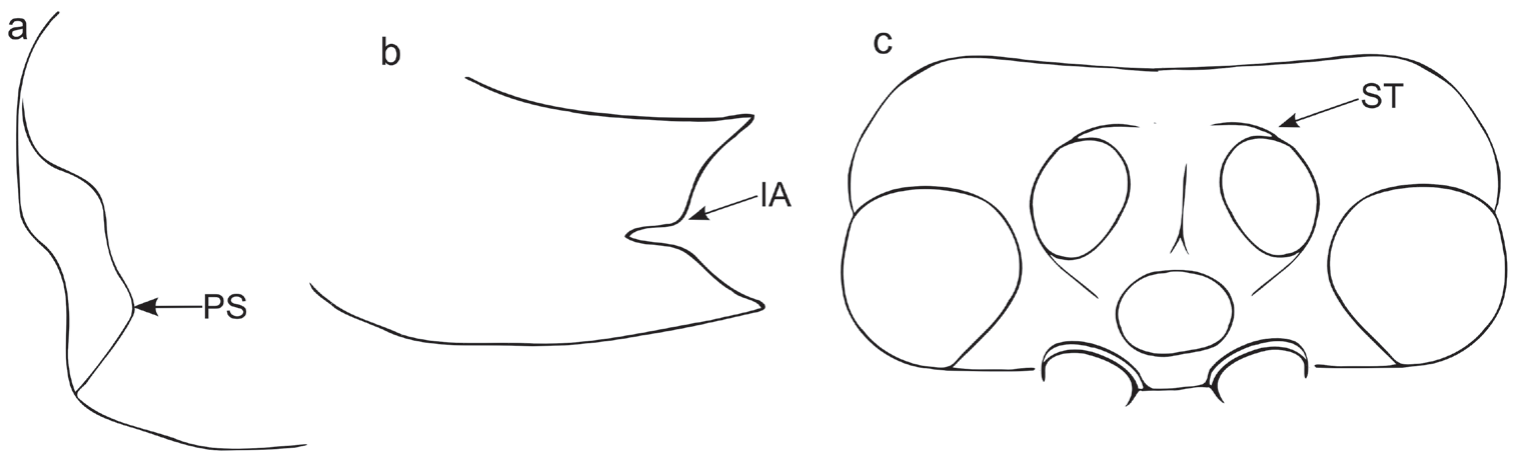
Morphological characters used in the species descriptions of the *Ophion scutellaris* species group that are not illustrated in the Hymenoptera Anatomy Ontology (portal.hymao.org). **a** Epicnemial carina; PS = Pleurosternal angle **b** Mandible; IA = internal angle of tooth **c** Stemmaticum.

Wing characters used in this study are illustrated in Figure 1. We have also included four indices of the fore wing that have been used in other treatments of Ophioninae (e.g. Gauld 1988). These indices are the alar index (AI), cubital index (CI), intercubital index (ICI), and the second discoidal index (SDI); these are defined in Table 1 and illustrated in Figure 1. The pleurosternal angle of the epicnemial carina is illustrated in Figure 8a (Brock 1982). The stemmaticum is the area containing the ocelli (Figure 8c). It is bounded by variously distinct sulci; when these sulci are strongly impressed and completely surround the stemmaticum, they are referred to as “complete” (Brock 1982).

**Figure 9.**
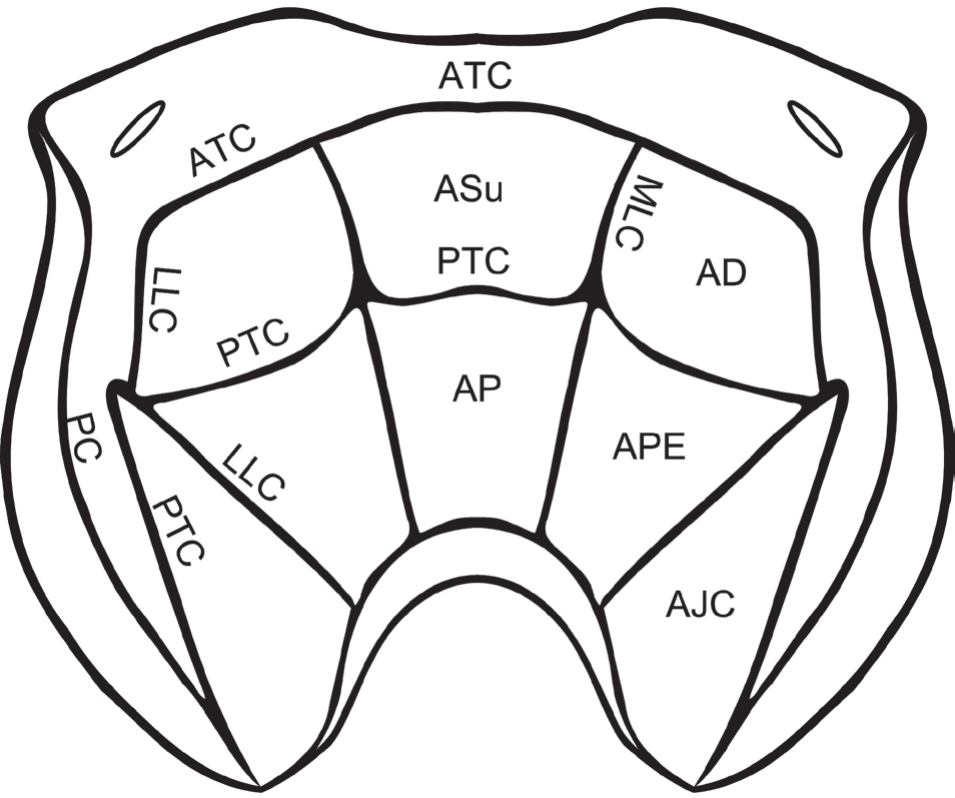
Propodeum of *Ophion* demonstrating propodeal carinae and areas. Carinae: ATC = anterior transverse carina; PTC = posterior transverse carina; MLC = median longitudinal carinae; LLC = lateral longitudinal carina; PC = pleural carina; Areas: ASu = area superomedia; AP = area petiolaris; AD = area dentiparis; APE = area postero-externa; AJC = area juxta-coxalis.

The carinae of the propodeum are often considered too highly variable to be of much use in species delimitation (Brock 1982, Gauld 1991). At least within this species group, however, we have found them to be quite useful. While they are certainly variable, particularly in the extent to which the various carinae are developed, there are usually at least a few essential elements that are consistent within species. Propodeal carinae and areas follow Gauld (1991) (Figure 9). The posterior area of the propodeum is the area posterior to the anterior transverse carina, while the spiracular area is anterior to the anterior transverse carina (Gauld 1988).

Other important characters are the sculpture of the face and clypeus, punctation of the metapleuron, number of flagellomeres, and overall size and colour.

### Key to the known Canadian species of the *Ophion scutellaris* species group

**Table:** 

1a	Face and most of thorax black, central flagellomeres approximately quadrate (Plate 7)	***Ophion dombroskii* Schwarzfeld, sp. n.**
1b	Body entirely orange or reddish, central flagellomeres distinctly longer than wide	**2**
2a	Anterior transverse carina weakly convex in centre (Figure 6a, 10a–c)	**3**
2b	Anterior transverse carina strongly convex in centre (Figure 6b, 10d–g)	**5**
3a	Fore wing length greater than 16 mm; antennae with at least 65 flagellomeres, more in female (Plate 1)	***Ophion keala* Schwarzfeld, sp. n.**
3b	Fore wing length less than 14 mm; antennae with less than 58 flagellomeres	**4**
4a	Median longitudinal carinae of propodeum strongly convergent posterior to areola, usually arriving at apex of propodeum as a single carina (Figure 10b); ramellus almost always extremely short to absent (rarely longer) (Figure 11b; Plate 2)	***Ophion idoneus* Viereck**
4b	Median longitudinal carinae of propodeum represented by wrinkles posterior to posterior transverse carina, convergent but not fusing into a single carina (Figure 10c); ramellus long (Figure 11c; Plate 3)	***Ophion importunus* Schwarzfeld, sp. n.**
5a	Yellow-orange base colour, wing length greater than 14 mm, propodeal carinae generally reduced, greatly reduced in male, but apical part of lateral longitudinal carinae expanded as a flange (Figure 10e; Plate 5)	***Ophion aureus* Schwarzfeld, sp. n.**
5b	Reddish-orange base colour, wing length 14 mm or less, propodeal carinae more developed, apical part of lateral longitudinal carinae strong but not expanded into a flange (Figure 10d, f)	**6**
6a	Facial punctures strong and separated by less than their diameter; orbits yellowish, stemmaticum and mesopleural fovea concolourous with base colour (Plate 4)	***Ophion clave* Schwarzfeld, sp. n.**
6b	Facial punctures very shallow and separated by 2–3× their diameter; orbits lacking yellow, concolourous with reddish head, stemmaticum and mesopleural fovea darker than base colour (Plate 6)	***Ophion brevipunctatus* Schwarzfeld, sp. n.**

### Species descriptions

#### *Ophion scutellaris* species group

**Diagnosis.** Most species are reddish-coloured, lacking yellow markings (except for narrowly on orbits, tegula, mesepimeron and rarely mandibles); early-season (most collected April to June); midtibial spurs nearly equal in length; eyes separated from posterior ocellus by 0.27–1.25× ocellar diameter; facial punctures more widely separated in centre of face than on sides; scutellum strongly carinate (at least partially); propodeum short, posterior area abruptly sloping so that it is nearly vertical at apex, anterior transverse carina (at least in centre) and lateral part of posterior transverse carina (along area juxta-coxalis) strong, otherwise carinae variable, but median longitudinal carinae always convergent apical of area superomedia; petiolar spiracle distinctly anterior to membrane of first metasomal segment.

**Description. Head:** Eyes convergent to nearly parallel, not strongly bulging (FW/HW: 0.46–0.56); eyes moderately indented (FW/FM: 0.69–0.91); face with minute to medium sized punctures, variously separated, but always closer together on sides and under toruli than in centre; clypeus coriaceous, variously punctate, CH/CAW: 0.49–0.70; mandible with internal angle well–defined (Figure 8b), punctate except for impunctate polished flange and tips; stemmaticum slightly to strongly raised, OC/OL: 0.42–1.2, OOD/OL: 0.11–0.92; IOD/OL: 0.27–1.25; ocellar carina rounded, in some species very slightly rippled or with a small peak at the apex; temple receding, usually approximately equal to width of eye in lateral view; antennae with 51–73 flagellomeres.

**Mesosoma:** Epicnemial carina with pleurosternal angle obtuse (rarely 90°); propodeum short, posterior area abruptly sloping so that it is nearly vertical at apex, ATC strong and convex along ASu, usually entirely strong, but sometimes obsolete or absent laterally, lateral part of PTC (along AJC) strong, usually absent or obsolete along APE, obsolete to strong along ASu; MLC variously developed, but always convergent apical of ASu, LLC mostly absent except strong to absent along AJC; PC strong, rarely connected to spiracle by a very weak carina, but usually not connected.

**Wings:** Wing L: 8.4–19.7 mm; CI: 0.39–0.84, AI: 1.13–3.31, SDI: 1.03–1.31, ICI: 0.44–1.00, *Rs* sinuate, ramellus absent to somewhat long, fenestra not extending below prestigma, lacking glabrous area in discocubital cell along *Rs+M*.

**Legs:** CL/CW: 1.41–2.37; trochantellus dorsally shorter than width; FL/FW: 6.3–11.6; midtibial spurs nearly equal in length (MTS: 0.70–0.97, usually > 0.80).

**Metasoma:** First metasomal segment with spiracles distinctly anterior to membrane; first metasomal segment 1.3–1.6× as long as second; second metasomal segment 2.4–4.5× as long as high.

**Colour:** Uniformly reddish, without yellow markings except orbit (usually narrowly), tegula, mesepimeron and rarely mandibles; females with ovipositor sheath concolourous with apex of metasoma; one species with head and mesosoma predominately black.

**Seasonality.** Early-flying species, most dates of capture in Canada are May to mid-June. Unusual dates of captures are one specimen that was collected in July, and one collected in August. At least in the northern Nearctic region, most other early-flying species can be distinguished from this species group by their distinct yellow markings.

**Biology.** There is virtually nothing known about the biology or hosts of the Nearctic species. The one exception is that *Ophion idoneus* has been recorded from the noctuid, *Sunira bicolorago* (Guenée) (Crumb 1924). The Palearctic species, *Ophion scutellaris* Thomsen, is a parasitoid of noctuids that overwinter as feeding larvae and are fully grown by spring (Brock 1982, G. Broad, pers. comm.). Since the Nearctic species are also early season parasitoids, they may have similar life-histories.

**Remarks.** The *Ophion scutellaris* species group can be further divided into two monophyletic subgroups (see text). One group (A) includes *Ophion idoneus*, *Ophion scutellaris*, *Ophion keala* and *Ophion importunus*. It can be recognized by the weakly arched anterior transverse carina of the propodeum (so the base of the area superomedia is only slightly convex; Figure 6a) and the more convex clypeus, distinctly separated from the face by a relatively strongly impressed epistomal sulcus, with smaller, denser, more regularly distributed punctures (Figure 7a–c). The second group (B) includes *Ophion clave*, *Ophion aureus*, *Ophion brevipunctatus*, and *Ophion dombroskii*, and can be recognized by the strongly arched anterior transverse carina of the propodeum (so that the base of the area superomedia is U-shaped; Figure 6b) and the flatter clypeus, weakly separated from the face by a less distinct epistomal sulcus, usually with coarse, irregularly scattered clypeal punctures (Figure 7d–f).

Only a small proportion of collected *Ophion* will generally be from the *Ophion scutellaris* species group. Within the group, *Ophion idoneus* is by far the most common Nearctic species.

#### Ophion keala

Taxon classificationAnimaliaHymenopteraIchneumonidae

Schwarzfeld sp. n.

http://zoobank.org/A4509217-2C71-4FB4-AED4-D81BF3344DA5

**Figure 10.**
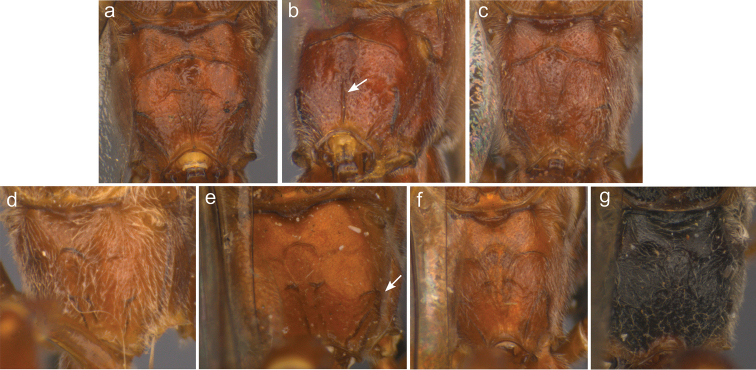
Propodeal carinae of species from the *Ophion scutellaris* species group. All images are of female specimens; male specimens have the same basic arrangement, but often with weaker carinae. **a** *keala* **b** *idoneus* (arrow indicates fused carinae) **c** *importunus* **d** *clave* **e** *aureus* (arrow indicates expanded, flange-like carina) **f** *brevipunctatus* **g** *dombroskii*.

**Figure 11.**
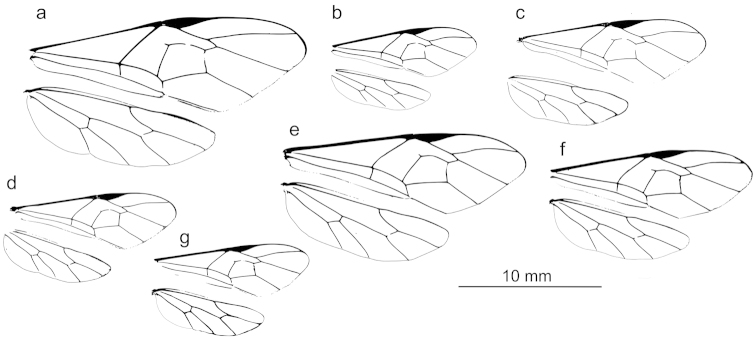
Representative fore wings and hind wings of species from the *Ophion scutellaris* species group. **a** *keala* **b** *idoneus* **c** *importunus* **d** *clave* **e** *aureus* **f** *brevipunctatus* **g** *dombroskii*.

Figures 10a , 11a ; Plate 1

##### Type material.

Holotype: ♀ (MS2249, DNA3965, GenBank KF594539, KF615948) CAN: AB: Porcupine Hills, Skyline Rd; 49.972 -114.087; 29 v 2008; UV trap; J.Dombroskie, J.Walker (CNC)

Paratypes: 7 ♀♀, 1 ♂. CAN: AB: 2 ♀♀ (MS2244, DNA3980, GenBank KF594552, KF615947; MS2238, DNA3960, GenBank KF594534, KF615950) Same data as holotype (CNC); 1 ♂, 1 ♀ (MS2235, DNA3904, GenBank KF594480, 615945; MS2237) Same data as holotype except date 28 v 2008 (CNC); 1 ♀ (MS8647, DNA6515, GenBank KF594730, KF615943, KF616332) Porcupine Hills, Skyline Rd, 49.972 -114.087, 15 vi 2009, UV trap J.Dombroskie, B.Brunet (CNC); 1 ♀ (MS7801, DNA7327, GenBank KF594917, KF615946) 62 km WNW of Dixonville, Mixedwood retention patch in clearcut, Site 7, 56.685 -118.641, 26 v 2008, UV trap, B.Bodeux (CNC); 1 ♀ (MS7912, DNA7324, GenBank KF594914, KF615944) 62 km WNW of Dixonville, Mixedwood retention patch in clearcut, Site 4, 56.684 -118.644, 7 vi 2008, UV trap, B.Bodeux (CNC); 1 ♀ (MS7746) 54 km NW of Dixonville, Mixedwood forest, 56.86 -118.31, 11 vi 2007, UV trap, B.Bodeux (CNC).

##### Etymology.

The name for this species is derived from *keala*, the Hawaiian word for path, as this large and distinctive species presents a rare clear path within the morphologically homogeneous jungle that is *Ophion*. It is a noun in apposition - and is also the name of the species author’s daughter.

##### Diagnosis.

♀: Wing L: 18.6–19.7 mm, Flag: 69–73; ♂: Wing L: 16.2 mm, Flag: 65; Very large species, uniformly dark reddish-orange with interocellar area often darker; hind femur long and slender (FW/FH: 8.4–11.6), scutellum strongly carinate.

##### Description.

**Head:** *Female*: Eyes convergent in frontal view; stemmaticum weakly raised, sulci surrounding stemmaticum complete; IOD/OL: 0.27–0.44; OOD/OL: 0.11–0.22; occipital carina rounded, often very slightly wavy, with a very small peak in the centre; OC/OL: 0.42–0.68; temple receding, approximately equal to width of eye in lateral view; CH/CAW: 0.52–0.63, coriaceous with evenly distributed medium-sized punctures, separated by approximately 1–2× their diameter, punctures smaller basally; face weakly coriaceous, with small punctures separated by 1–2× their diameter, closer on sides than in centre; FW/FH: 1.24–1.49; antennae with 69–73 flagellomeres; F1: 3.56–4.50; F20: 1.54–2.24; MS/MW: 0.33–0.47; GI/MW: 0.46–0.69; *Male*: Same, except: Eyes more weakly convergent, OOD/OL: 0.44; OC/OL: 0.81; flagellomeres: 65.

**Mesosoma:** Mesoscutum polished, evenly punctate with minute punctures separated 1–2× their diameter; mesopleuron coriaceous, densely punctate with small to medium-sized relatively shallow punctures, separated by approximately their diameter; subpolished with minute punctures separated by 2–3× their diameter above mesopleural fovea; epicnemial carina with pleurosternal angle obtuse, distinctly so in male, rounded (see variation); SL/SW: 1.47–1.76, lateral carinae strong along almost entire length of scutellum, slightly weaker in male; metapleuron strongly coriaceous, punctures approximately equal to those of posterior corner of mesopleuron, equally or slightly less dense.

**Propodeum:** ATC strong, very slightly arched above ASu; PTC weak to absent along ASu, represented by small crests at MLC, obsolete to absent along AD, very strong along AJC where it is expanded as a flange; MLC obsolete, absent to very faintly represented by parallel or slightly convergent wrinkles along ASu, present as a series of stronger convergent wrinkles along AP; LLC weak to obsolete along AJC, otherwise absent; PC strong, connected to spiracle by a very weak to obsolete carina; spiracular area sloping, subpolished with minute shallow punctures separated by 1–2× their diameter; posterior area weakly wavy-wrinkled with minute punctures to PTC, then more distinctly wrinkled to apex.

**Wings:** *Female*: Wing L: 18.6–19.7 mm, CI: 0.54–0.70, AI: 1.42–2.01, SDI: 1.13–1.27, ICI: 0.61–0.93; wings slightly brownish with black veins, stigma reddish-brown, ramellus very short to somewhat long, fenestra restricted to area under stigma; *Male*: Same, except wing L: 162 mm.

**Legs:** *Female*: CL/CW: 1.94–2.37; FL/FW: 8.41–11.63, MT1/MT2: 2.06–2.33; MTS: 0.78–0.88 (♀); *Male*: Same, except CL/CW: 1.83; MTS: 0.91.

**Metasoma:** First metasomal segment often abruptly (sometimes gradually) expanded from petiole to postpetiole, spiracles sometimes raised on tubercles.

**Colour:** Reddish-orange; stemmaticum often slightly darker, mandibles usually slightly paler; orbit (especially posterior to eye), tegula and dorsal part of mesepimeron yellowish; notauli and margins of mesocutum slightly darker.

##### Variation.

One ♀ with all punctures slightly more sparsely distributed than in the remaining specimens; One ♀ with pleurosternal angle of epicnemial carina approximately 90° and somewhat sharp.

##### Seasonality.

All collections have been from late May until mid-June.

#### Ophion idoneus

Taxon classificationAnimaliaHymenopteraIchneumonidae

Viereck, 1905

Figures 10b , 11b ; Plate 2

##### Material examined.

Holotype: ♂ USA: Kansas: Douglas Co. ft. 900; May; U. of K., Lot 878, 8ub (SEMC)

Other material: 109 ♀♀, 83 ♂♂, 2 unknown: CAN: AB: 2 ♀♀ (MS12403, MS12405) 11 km NE of Lacombe, J.J. Collett Natural Area, N-facing slope, P. glauca, B. papyrifera, 835m, 52.553 -113.641, 18 vi 2009, UV, C.D. Bird (CNC); 4 ♀♀, 1 ♂ (MS11824–28) 5km NEE of Dunstable, George Lake Research Station, aspen forest, MT-7, 53.957 -114.130, 25-29 v 2007, Malaise, M. Schwarzfeld (UASM); 11 ♀♀, 6 ♂♂ (MS13317, MS13319–20, MS13322–26, MS13328–36) same data except date is 29 v-6vi 2007 (CNC); 2 ♀♀, 6 ♂♂ (MS12920, MS12922–28) 5km NEE of Dunstable, George Lake Research Station; Black spruce forest; MT-8, 53.957 -114.128, 29 v-6 vi 2007, Malaise, M. Schwarzfeld (UASM); 4 ♀♀ (MS12433–35, MS12444) 8 km NW of Winfield, Bird East Poplar Creek quarter, mixed woods, 900m, 53.01 -114.50, 12 vi 2010, UV, C.D. Bird (CNC); 1 ♂ (MS12335) same data except date is 15 v 2010 (UASM); 1 ♂ (MS11457) same data except date is 16 v 2009 (UASM); 1 ♀ (MS13925) same data except date is 21 v 2011 (UASM); 1 ♀ (MS2261) 8 km SE Sherwood Park, aspen forest, 53.478 -113.229, 12-15 v 2008, MV light, G.R. Pohl (UASM); 1 ♀ (MS7373) Bragg Creek, 50.917 -114.533, 15 viii 2007, at light, F. Sperling (CNC); 1 ♀ (MS4026), Calgary, Edgemont, 51.115 -114.142, 18 v 2010, light, T. Pike (CNC); 3 ♀♀ (MS2275, DNA3910, GenBank KF594486, KF615953; MS2280; MS2274) Edmonton, nr. Fulton Ravine, 53.545 -113.439, 15-16 v 2008, light, G. Anweiler (CNC); 1 ♂ (MS2284) same data except date is 17 v 2008 (UASM); 1 ♀ (MS9688, DNA3974, GenBank KF594547, KF615956) same data except date is 23 v 2009 (CNC); 2 ♀♀ (MS2286, MS2290) same data except date is 24 v 2008 (UASM); 1 ♀, 1 ♂ (MS5632, DNA3933, GenBank KF594507, KF615951; MS4301) same data except date is 26 v 2011 (CNC); 9 ♀♀ (MS13809, MS13811, MS13815, MS13820-22, MS13826-28) same data except date is 27 v 2011 (UASM); 3 ♀♀ (MS12169, MS12171–72) same data except date is 31 v 2009 (UASM); 7 ♀♀ (MS13834–35, MS13842–43, MS13858, MS13862) same data except date is 6-8 vi 2011 (UASM); 5 ♀♀, 2 ♂♂ (MS13763–65, MS13767, MS13757, MS13774, MS13782) same data except date is 9-11 vi 2011 (UASM); 2 ♂♂ (MS78, MS80) same data except date is v 2007 (CNC); 2 ♀♀ (MS2879, MS2886) Edmonton, Edith Ravine, 53.510 -113.622, 12 v 2010, UV light, J. Acorn (UASM); 1 ♀ (MS2885) same data except date is 27 v 2010 (UASM); 1 ♀ (MS2234, DNA5552, GenBank KF594576, KF615958) Edmonton, inside building, 53.521 -113.521, J. Dombroskie (CNC); 3 ♂♂ (MS100, MS103, MS107) Edmonton, River Valley at U.Alberta, 53.529 -113.519, 28 v 2007, Sweep, M. Schwarzfeld (CNC); 1 ♀ (MS2770, DNA6551, GenBank KF594763, KF615949) EMEND site, 48 km NW of Dixonville; Decid. forest, uncut, 56.7525 -118.3282, 28 v-10 vi 2008, Malaise, 852-2, M. Schwarzfeld (CNC); 2 ♀♀ (MS12420, MS12423) Erskine, 5 Maple Close, backyard with aspen, 830m, 52.322 -112.883, 19 v 2010, UV, C.D. Bird (UASM); 1 ♀, 1 ♂ (MS9763–64) George Lake Research Site, 53.953 -114.120, 29 v 2007, Sweep, M. Schwarzfeld (UASM); 1 ♂ (MS39) George Lake Research Site, Aspen forest, 53.957 -114.125, 25 v 2007, Sweep, M. Schwarzfeld (UASM); 1 ♀ (MS3613) N. Wyndham-Carseland Provincial Park, 50.8366 -113.4347, 31 v 2008, light, T. Pike (UASM); 2 ♀♀ (MS5650, DNA3977, GenBank KF594549, KF615957; MS5651, DNA3950, GenBank KF594524, KF615952) Pigeon Lake, Itaska, 53.072 -114.072 3 vi 2008, UV light, F. Sperling (CNC); 1 ♀ Pigeon Lake, Itaska, May 28, 2006, F.A.H. Sperling coll.; 9 ♀♀ 14 ♂♂ (MS12460, MS12463, MS12472, MS12474–83, MS12485–93, MS12495) Rochon Sands Prov. Park, 15 km N Erskine, 720m, aspen, chokecherry, aspen, 52.46 -112.88, 2 vi 2010, UV, C.D. Bird (UASM); 1 ♂ (MS12197) Spruce Grove, 13 km South, 53.4 -113.9, 28 v-2 vi 1989, Malaise, A.T. Finnamore (CNC); 1 ♀ (MS12265) Summer Village of Gull Lake, 52.460 -113.947, 31 v 2009, UV trap, J.H. Acorn (UASM); 1 ♂ (MS13966) Wintering Hills West, 51.2552 -112.6261, 29 v 2011, net, J. Dupuis (UASM); BC: 1 ♂ (ENT008-002270) Robson, “?” v 1951, H.R. Foxlee, Ex. H.R. Foxlee Collection U.B.C. 1971 (RBCM); 1 ♂ (ENT008-005274) same data except date is 14 v 1954 (RBCM); 1 ♀ (ENT008-002250) same data except date is 15 v 1951 (RBCM); 1 ♂ (ENT008-002274) same data except date is 15 v 1954 (RBCM); 1 ♀, 1 ♂ (ENT008-002252, ENT008-002275) same data except date is 17 v 1954 (RBCM); 1 ♂ (ENT008-002230) same data except date is 29 v 53 (RBCM); 1 ♂ (ENT008-002239) same data except date is 3 v 51 (RBCM); 1 ♂ Robson, 8 v 1954, H.R. Foxlee (UBCZ); MB: 2 ♀♀ 10 mi. S. of Winnipeg, 1 vi 1973, coll. C. Starr, at UV light (SEMC); 2 ♀♀ same data except date is 30 v 73 (SEMC); 1 ♀ Brandon, 29 v 49, Light Trap (NFRC); ON: 4 ♀♀, 2 ♂♂ (MS2927, MS2932–34, MS2940, MS2943) Bells Corners, Monaghan Forest, 45.272 -75.808, 18 v 2010, Light, B.C. Schmidt (UASM); 1 ♀, 1 ♂ (MS8020, MS8039) Bells Corners, Stony Swamp, 45.295 -75.830, 25 v 2008, B.C. Schmidt (UASM); 1 ♂ (MS12164) Bells Corners, Stony Swamp, 45.295 -75.830, 3 v 2010, MV light, J. Dombroskie, B.C. Schmidt (UASM); 1 ♀, 1 unknown (MS10755, DNA5554, GenBank KF594578, KF615955; MS10754) Grenadier Is., St. Lawrence Islands Nat. Pk., *Carya* grove, 44.4 -75.9 10–21 vi 1994, YPT, Coll. CNC Hym Team (CNC); 1 ♀ (MS10710) same data except date is 24 v–9 vi 1994 (CNC); 7 ♂♂ (MS10713, DNA6552, GenBank KF594764, KF615960; MS10711; MS10716–17; MS10719–21) same data except trap is Malaise, date is 24 v–9 vi 1994 (CNC); 1 ♀ (MS10751) same data except trap is Malaise, date is 3–13 v 1994 (CNC); 1 ♀ (MS8007, DNA3976, GenBank KF594548, KF615961) Leeds Grenville Co., Long Mtn. 44.487 -76.008, 7 vi 2008, B.D. Schmidt (CNC); 2 ♀♀ (MS10807, MS10813) same data except date is 21 v 2009 (UASM); 5 ♀♀, 22 ♂♂ (MS10793, DNA5717, GenBank KF594635, KF615959; MS10794, DNA5552, GenBank KF594576, KF615958; MS10779, DNA5551, GenBank KF594575, KF615954; MS10768–78, MS10780–92) Ottawa, city garden, 45.356 -75.707, 5 v-5 vi 2008; Malaise, Coll. H. Goulet (CNC); 1 ♂ Waterloo Co., Cambridge, malaise, Skevington & Cannings (RBCM); SK: 1 ♀ S’toon, May 22, 1940, D.R. Foskett (RBCM); USA: MI: 1 ♀ Ag. Coll. Mich 5–23 93 22 (CUIC); 1 ♂ Ag. Coll. Mich 5-23 95 22 (CUIC); NY: 1 ♀, 1 ♂ Ithaca, v 23 1936 (CUIC); 1 unknown Orient, L.I. June 2, 1932, Roy Latham/ Roy Latham Collection (CUIC).

##### Diagnosis.

Smallest species within the species group; MLC fused immediately apically of ASu, therefore appears Y-shaped; ramellus usually absent or extremely short; Wing L: 8.4–11.5 mm, Flag: 51–57.

##### Description.

**Head:** Eyes weakly convergent in frontal view; stemmaticum very weakly raised, sulci not complete; IOD/OL: 0.58–1.25, OOD/OL: 0.25–0.43 (♀), 0.35–0.92 (♂); occipital carina rounded, often very slightly rippled or wavy, usually with a small peak at centre, OC/OL: 0.58–0.95; temple receding, approximately equal to eye width in lateral view; clypeus convex in lateral view and distinctly separated from face, coriaceous, with small regular shallow punctures only slightly larger than on face, separated by approximately their diameter, CH/CAW: 0.57–0.70; face subpolished to very weakly coriaceous, with small shallow punctures separated by approximately their diameter, slightly more dense on sides; FW/FH: 1.28–1.45; antennae with 51–57 flagellomeres, F1: 2.93–4.00; F20: 1.33–2.00; MS/MW: 0.50–0.82; GI/MW: 0.46–0.73.

**Mesosoma:** Mesoscutum subpolished with very small to minute regular punctures separated by approximately 2× their diameter; mesopleuron weakly coriaceous, strongly closely punctate, punctures separated by their diameter ore less, above mesopleural fovea subpolished with small punctures separated by 1–3× their diameter; metapleuron slightly more coriaceous, punctures similar in size to those of mesopleuron, only slightly less dense; epicnemial carina with pleurosternal angle obtuse; scutellum with lateral carina strong along most of length, SL/SW: 1.21–1.69.

**Propodeum:** ATC strong, weakly arched above ASu; PTC weak to obsolete along ASu, raised into small crests where intersects with MLC, absent along AD, strong along AJC; MLC obsolete and weakly convergent along ASu, strongly convergent just apical to ASu, so that the carinae fuse into one stronger carina for most of AP; LLC almost absent, short carina in AJC may be remnant of this carina; PC strong, not connected to spiracle; spiracular area slightly sloping, nearly horizontal, subpolished with shallow minute indistinct punctures, separated by 2–3× their diameter; posterior area wavy-wrinkled with very shallow indistinct punctures basally, more wrinkled apically.

**Wings:** Wing L: 8.4–11.5 mm, CI: 0.48–0.84, AI: 1.95–3.31, SDI: 1.03–1.29, ICI: 0.44–0.80; wings with veins dark brown, stigma light brown with apex whitish-gray, ramellus completely absent or represented by a minute vestigial stub, rarely somewhat long, fenestra not extending below prestigma.

**Legs:** CL/CW: 1.41–1.85, FL/FW: 6.31–7.85, MT1/MT2: 2.09–2.50; MTS: 0.71–0.89.

**Metasoma:** Expansion from petiole to postpetiole gradual.

**Colour:** Reddish-orange, mandibles, palps and legs slightly paler; orbits, tegula and dorsal part of mesepimeron yellowish.

##### Seasonality.

All collection records are from May and June, with the exception of a single specimen collected on Aug 15 from Bragg Creek, Alberta.

##### Remarks.

Common and wide-spread early-season species. Most small dark reddish individuals, lacking yellow notauli, collected in May will be this species, at least in the northern Nearctic region. The original description (Viereck 1905) for this species is quite detailed, and is sufficient to identify this species; I have re-described it here so that the description is consistent with the other members of the species group. I excluded characters from the original description that apply to the species group as a whole, and focused on characters that distinguish it from other species within the group.

#### Ophion importunus

Taxon classificationAnimaliaHymenopteraIchneumonidae

Schwarzfeld sp. n.

http://zoobank.org/284591E3-6786-44DB-AF14-CDC0767ACF9C

Figures 10c , 11c ; Plate 3

##### Type material.

Holotype: ♀ (MS12343, DNA6907, GenBank KF594814, KF615963, KF616346) CAN: AB: 8 km NW of Winfield, Bird East Poplar Creek quarter, mixed woods, 900m; 53.01 -114.5; 15 v 2010; UV; C.D.Bird (CNC).

Paratypes: 2 ♀♀. CAN: AB: 1 ♀ (MS13904) 8 km NW of Winfield, Bird East Poplar Creek quarter, mixed woods, 900m, 53.01 -114.5, 21 v 2011, UV, C.D.Bird (CNC). ON: 1 ♀ (MS12153) Bells Corners, Stony Swamp, 45.295 -75.83, 3 v 2010, MV light, J.Dombroskie, B.C.Schmidt (CNC).

##### Etymology.

This species is most similar to *Ophion idoneus*. Since *idoneus* is the Latin word for “suitable” or “proper”, the name for this species is derived from the Latin word *importunus*, meaning “unsuitable”.

##### Diagnosis.

Similar to *Ophion idoneus*, but can be recognized by the larger size, LMC convergent but not meeting (thus propodeum lacks Y-shaped carinae), and the long ramellus. Wing L: 12.6–13.3 mm, Flag: 54–57.

##### Description.

**Head:** Eyes weakly convergent in frontal view; stemmaticum weakly raised, sulci not complete; IOD/OL: 0.53–0.85, OOD: 0.15–0.23; occipital carina rounded, very slightly rippled or wavy, OC/OL: 0.80–1.00; temple receding, approximately equal to eye width in lateral view; clypeus convex, weakly coriaceous, with small regular punctures only slightly larger than on face, separated by approximately their diameter, CH/CAW: 50–0.60; face subpolished with very small punctures separated by slightly more than their diameter in the centre and slightly less on the sides; FW/FH: 1.25–1.29; antennae with 54–57 flagellomeres, F1: 3.43–3.69; F20: 1.54–1.83; MS/MS: 0.46–0.58; GI/MW: 0.46–0.54.

**Mesosoma:** Mesoscutum subpolished with very small regular punctures separated by 1–2× their diameter; mesopleuron and metapleuron coriaceous, strongly closely punctate, punctures separated by less than their diameter; area of mesopleuron above mesopleural fovea subpolished with minute punctures separated by 1–3× their diameter; epicnemial carina: pleurosternal angle obtuse, rounded; scutellum with lateral carina strong along most of length, SL/SW: 1.55–1.60; **Propodeum:** ATC strong, moderately arched above ASu; PTC present along AJC, otherwise absent; MLC very weak and weakly convergent along ASu, stronger at apex of ASu, obsolete and represented by strongly convergent wrinkles along AP; LLC represented by indistinct wrinkles along AJC, otherwise absent; PC strong, sometimes weakly connected to spiracle by an obsolete carina; spiracular area sloping, subpolished with small punctures separated by less than their diameter; posterior area wavy-wrinkled with very shallow punctures basally, more wrinkled apically.

**Wings:** Wing L: 12.6–13.3 mm, CI: 0.60–0.67, AI: 1.58–1.89, SDI: 1.06–1.22, ICI: 0.62–0.80, wings with veins black, stigma reddish-brown with apex whitish-gray, ramellus long, fenestra not extending below prestigma.

**Legs:** CL/CW: 1.61–2.00, FL/FW: 8.25–8.87, MT1/MT2: 2.19–2.26; MTS: 0.79–0.87.

**Metasoma:** Expansion from petiole to postpetiole somewhat abrupt.

**Colour:** Reddish-orange, orbits and sometimes mandibles yellow, tegula and dorsal part of mesepimeron yellowish, scutellum slightly paler than base colour.

##### Seasonality.

All three collection records are in May.

#### Ophion clave

Taxon classificationAnimaliaHymenopteraIchneumonidae

Schwarzfeld sp. n.

http://zoobank.org/59F09AD9-0EDE-490F-BA1A-A84128CB9ED4

Figures 10d , 11d ; Plate 4

##### Type material.

Holotype: ♀ (MS12193, DNA6944, GenBank KF594831, KF615972, KF616352): CAN: AB: Spruce Grove, 13 km South, 53.4 -113.9, 28 v-2 vi 1989, Malaise, A.T.Finnamore (CNC)

Paratypes: 13 ♀♀, 3 ♂♂. CAN: AB: 2 ♀♀(MS12198–99) Same data as holotype (CNC); 1 ♂, 3 ♀♀ (MS13746, DNA7383, GenBank KF594963; MS13749, DNA7381, GenBank KF594961; MS13748; MS13752) Jenner Bridge, S. Jenner, riparian willow/sagebrush shrub, 50.844 -111.154, 2 vi 2010, UV trap, G.Anweiler (CNC); 1 ♀ (MS1689, DNA7391, GenBank KF594970) Jenner rodeo grounds, 50.842 -111.151, 07 vi 2007, Light, G.Anweiler (CNC); 1 ♂, 1 ♀ (MS71, DNA7382, GenBank KF594962; MS72) Jenner rodeo grounds, 50.842 -111.151, 09 v 2007, UV trap, M.Schwarzfeld (CNC); 1 ♀ (MS1434) Jenner rodeo grounds, 50.842 -111.151, 26 v 2007, UV trap, J.Dombroskie & G.Anweiler (CNC); 1 ♀ (MS12386) 17 km S of Stettler, Lowden Springs Conserv. Area, aspen/buckbrush/grassland, 822m, 52.154 -112.713; 26 v 2010; UV; C.D.Bird (CNC); 1 ♂ (MS11578) 17 km S of Stettler, Lowden Springs Conserv. Area, native prairie, 825m, 52.154 -112.712, 24 v 2009, UV, C.D.Bird (CNC). MB: 1 ♀ Brandon, 29.v.49, light trap (NFRC); ON: 2 ♀♀ (MS10758, MS10763) Leeds, Grenville Co. Long Mtn, 44.487 -76.008, 27 iv 2009, B.C.Schmidt (CNC); 1 ♀ Waterloo Co., Cambridge, malaise, 18-21.v.1992, Skevington & Cannings (RBCM)

##### Etymology.

“*Clave*” is the Spanish word for key, and refers to the arrangement of the propodeal carinae, which resemble an old-fashioned keyhole. It is also the fundamental rhythm in salsa music, which was undoubtedly playing as this species was being described. It is a noun in apposition.

##### Diagnosis.

ATC of propodeum U-shaped above ASu; stemmaticum raised; occipital carina rounded; face coarsely densely punctate (most punctures separated by less than their diameter), and punctures connected by strong microreticulation; Wing L: 10.5–12.4 mm; Flag: 52–60. This species is most similar to *Ophion brevipunctatus*, but can be distinguished by the sculpture of the face, the more extensive yellow orbits and the smaller size. This species is also similar to *Ophion idoneus* and *Ophion importunus*, but can be most easily distinguished by the strongly convex ATC.

##### Description.

**Head:** Eyes slightly convergent to nearly parallel in frontal view; stemmaticum raised, sulci surrounding stemmaticum complete or nearly so; IOD/OL: 0.53–1.00; OOD/OL: 0.13–0.29; occipital carina rounded, OC/OL: 0.67–0.92; temple receding, approximately equal to width of eye in lateral view; clypeus weakly convex in lateral view and very weakly separated from face, coriaceous with irregularly scattered coarse punctures, often interspersed with at least a few minute punctures, smaller and denser towards lateral and dorsal margins, less dense than on face (see variation), CH/CAW: 0.49–0.67; face evenly coarsely punctate, most punctures separated by less than their diameter and connected by strong microreticulation, smaller on sides than in centre (see variation); FW/FH: 1.18–1.26; antennae with 52–60 flagellomeres, F1: 3.00–4.17, F20: 1.36–1.83; MS/MW: 0.43–0.63; GI/MW: 0.36–0.67

**Mesosoma:** *Female*: Mesoscutum polished to subpolished (see variation), evenly, shallowly punctate with small to minute punctures; mesopleuron coriaceous with strong punctures separated by approximately their diameter, polished with sparse minute punctures above mesopleural fovea; epicnemial carina of females with pleurosternal angle approximately 90°, and more or less sharp, varying from slightly acute to slightly obtuse; SL/SW: 1.23–1.58; lateral carina strong along anterior third to two-thirds of scutellum; metapleuron coriaceous with strong punctures separated by approximately their diameter; *Male*: Same, except SL/SW: 1.43–1.67; pleurosternal angle of epicnemial carina rounded and slightly obtuse.

**Propodeum:** *Female*: ATC strong, strongly arched above ASu (so anterior margin of ASu is highly convex), occasionally weak to obsolete along AE; PTC present (occasionally obsolete) along ASu, usually produced as small crests where intersects with MLC, obsolete to absent between AD and APE, strong along AJC; MLC weak and slightly convergent along ASu, strong (occasionally weak) and distinctly convergent (but not meeting) along AP; LLC weak to strong along AJC, otherwise absent; PC strong, not connected to spiracle; spiracular area slightly to strongly sloping, closely punctate, subpolished to coriaceous; posterior area rugulopunctate basally, strongly wrinkled apically; *Male*: Same pattern but all carinae tending to be weaker.

**Wings:** Wing L: 10.5–12.4 mm, CI: 0.39–0.59, AI: 1.13–2.16, SDI: 1.16–1.30, ICI: 0.52–0.93; veins dark brown to black, stigma light brown; fore wing with ramellus absent or present, discocubital cell entirely trichiose except for well-defined fenestra under stigma (not extending below prestigma).

**Legs:** *Female*: CL/CW: 1.58–1.93; FL/FW: 7.00–8.59 (♀), 7.00–7.18 (♂), MT1/MT2: 2.01–2.26 (♀); MTS: 0.72–0.88; *Male*: Same, except MT1/MT2: 1.69–2.02.

**Metasoma:** Sides of petiole gently divergent from spiracles to apex in females, more abruptly expanded at spiracles in males.

**Colour:** Reddish-orange; palps, mandibles and/or scutellum sometimes slightly paler; orbits narrowly yellowish; tegula and mesepimeron yellowish.

##### Variation.

There is some geographic variation in this species, with less morphological variation in specimens from within each locality than there is between localities.

2 ♀♀ have clypeal punctures more densely and regularly spaced than in remaining specimens; 2 ♀♀ have facial punctures slightly smaller and facial microreticulation slightly weaker that in the remaining specimens; 1 ♂, 1 ♀ (from same locality) with mesoscutum coriaceous.

##### Seasonality.

This species has been collected from late April until early June.

#### Ophion aureus

Taxon classificationAnimaliaHymenopteraIchneumonidae

Schwarzfeld sp. n.

http://zoobank.org/A6525116-194C-42DC-B454-FC927FF00AB1

**Plate 5.**
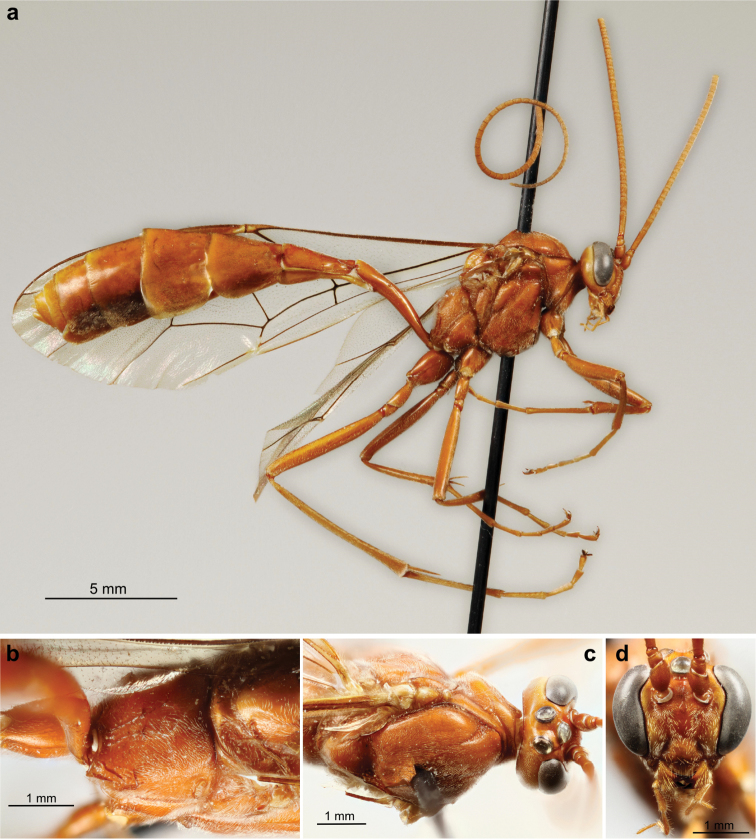
*Ophion aureus* Schwarzfeld sp.n., type specimen; **a** habitus **b** propodeum **c** mesothorax and head **d** face.

Figures 10e , 11e ; Plate 5

##### Type material.

Holotype ♀ (MS7632, DNA3970, GenBank KF615968) CAN: AB: Machesis Lk Forest Prov. Rec. Area, 32 km W Fort Vermilion, 318m, Jack pine forest, 58.325 -116.578, 10 vi 2008, UV trap, D.&S. Macaulay (CNC).

Paratypes 9 ♂♂ (MS2318, DNA3961, GenBank KF594535, KF645966; MS2324, DNA3911, GenBank KF594487, KF615965; MS2310, DNA3975, GenBank KF615969; MS2311, MS2313–16, MS2320) nr. Tangent Park Campgrnd, 23km S of Peace River, meadow in spruce/aspen, 56.092 -117.542; 7 v 2008, UV trap, D.Macaulay (CNC).

##### Etymology.

The name *aureus* is the Latin word for golden, referring to the golden-orange colour of this species.

##### Diagnosis.

Wing L: 14.3–17.1 mm, Flag: 65–69. Largest species in subgroup B; stemmaticum raised with sulci complete; reduced propodeal carinae with posterior area strongly wrinkled; more golden-orange (less reddish) than the other species in this species group.

##### Description.

**Head:** Eyes weakly convergent in frontal view; stemmaticum distinctly raised, sulci surrounding stemmaticum complete and deeply impressed; IOD/OL: 0.65–0.80, OOD/OL 0.26–0.33; occipital carina rounded, OC/OL: 0.73–1.00; temple receding, approximately equal to width of eye in lateral view; clypeus moderately convex in lateral view and weakly separated from face, slightly more convex in males, coriaceous, sparsely punctate in males, very sparsely punctate in female, with irregularly sized (minute to coarse) punctures, punctures denser basally and especially laterally, CH/CAW: 0.0.53–0.64; face coriaceous with small punctures separated by slightly more than their diameter, smaller and denser on orbits than in centre, FW/FH: 1.25–1.41; antennae with 65–69 flagellomeres, F1: 3.25–4.00; F20: 1.47–2.38; MS/MW: 0.52–0.76; GI/MW: 0.32–0.66.

**Mesosoma:** Mesoscutum densely evenly punctate, subpolished with minute punctures in males, weakly coriaceous with slightly larger punctures in female; mesopleuron coriaceous with strong punctures separated by approximately their diameter in female, punctures smaller and separated by approximately 2× their diameter in males, subpolished with smaller sparser punctures above mesopleural fovea, epicnemial carina with pleurosternal angle 90° to slightly obtuse, rounded (occasionally somewhat sharp); scutellum with lateral carina strong at base, present along basal third to half; SL/SW: 1.13–1.38; metapleuron coriaceous (strongly coriaceous in female), punctures smaller and sparser than on mesopleuron.

**Propodeum:** *Female*: ATC strong along ASu, strongly arched (so anterior margin of ASu is highly convex), otherwise absent; PTC present along ASu, raised into crests where intersects with MLC, absent along APE, extremely strong (raised as a flange) along AJC; MLC very weak and slightly convergent along ASu, stronger and nearly parallel along AP; LLC absent; two strong longitudinal wrinkles in AJC, one of which probably represents remnant of LLC; PC strong, not connected to spiracle; propodeum short, spiracular area sloping, punctate, strongly coriaceous, posterior area rugose; *Male*: similar, but with all carinae much less developed: ATC present as vestige in centre; PTC present only as slight crests where intersects with MLC and as a flange along AJC, but much less raised than in female; MLC convergent, weak to obsolete, often reduced to longitudinal wrinkles; punctures on spiracular area very shallow.

**Wings:** Wing L: 14.3–17.1 mm, CI: 0.41–0.60, AI: 1.57–2.36, SDI: 1.17–1.31, ICI: 0.70–1.00; veins brown to dark brown, stigma light brown with an apical white spot, fenestra mostly confined to area below stigma, in several specimens indistinctly extending below prestigma; trichiae more or less sparse along Rs+M; ramellus long

**Legs:** *Female*: CL/CW: 1.67, FL/FW: 7.95; MT1/MT2: 1.22; MTS: 0.86; *Male*: CL/CW: 1.69–2.08; FL/FW: 8.11–9.63; MT1/MT2: 1.22–2.19; MTS: 0.70–0.90.

**Metasoma:** Sides of petiole gently divergent from spiracles to apex, some males with more abrupt expansion at spiracles

**Colour:** Uniformly golden-orange, males less reddish than other members of the species group, female distinctly less reddish. Orbits narrowly and indistinctly yellow, more yellow posterior to eye; tegula and extreme dorsal part of mesepimeron yellowish.

##### Seasonality.

This species had been collected in only two collection events, the males in mid-May and the one female in mid-June.

#### Ophion brevipunctatus

Taxon classificationAnimaliaHymenopteraIchneumonidae

Schwarzfeld sp. n.

http://zoobank.org/88A11622-15EA-40E9-B988-1EAC4AF972E2

**Plate 6.**
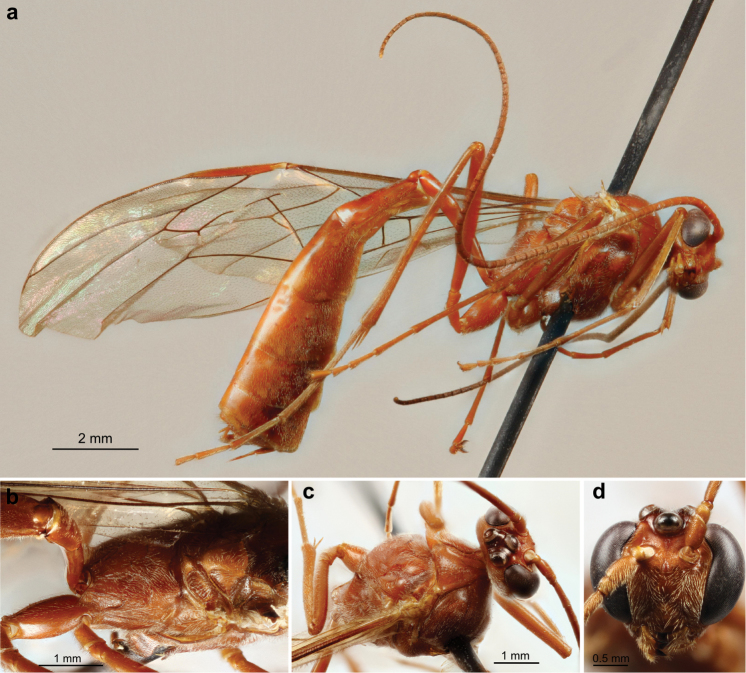
*Ophion brevipunctatus* Schwarzfeld sp.n., type specimen; **a** habitus **b** propodeum and scutellum **c** mesothorax and head **d** face.

Figures 10f , 11f ; Plate 6

##### Material examined.

Holotype ♀ (MS7990, DNA3939, GenBank KF594513, KF615967, KF616314) CAN: ON: Carleton Co., Carp Ridge, nr. Carp; 45.385 -76.008; 13 v 2008; UV light; B.C. Schmidt (CNC).

##### Etymology.

The name is derived from the Latin words *brevis* and *punctatus*, referring to the unusually shallow punctures of the face.

##### Diagnosis.

Wing L: 14.0 mm; Flag: 67; ATC strongly arched above ASu; stemmaticum raised with sulci complete; facial punctures small, very shallow, widely separated but connected with strong microreticulation; temple strongly receding, 0.6× eye width (other species in this group with temple approximately equal to eye width); stemmaticum dark, no yellow on orbits.

##### Description.

**Head:** Eyes convergent in frontal view; stemmaticum raised, sulci complete; IOD/OL: 0.69, OOD/OL: 0.13; occipital carina rounded, OC/OL: 0.78; temple strongly receding, 0.6× as long as eye width in lateral view; CH/CAW 0.58× apical width, only slightly convex in lateral view, weakly separated from face; clypeal punctures irregularly sized, sparsely, irregularly distributed across coriaceous clypeus; punctures of face small, very shallow, separated by 2–3× their diameter, connected by strong microreticulation; FW/FH: 1.27; antennae with 67 flagellomeres; F1: 3.6; F20: 1.8; MS/MW: 0.5; GI/MW: 0.5.

**Mesosoma:** Mesoscutum subpolished, evenly punctate with minute punctures; mesopleuron coriaceous with strong punctures separated by approximately their diameter, varying to subpolished with smaller punctures anteriodorsally; subpolished with minute punctures above mesopleural fovea; epicnemial carina with pleurosternal angle slightly obtuse; SL/SW: 1.5, strongly carinate along the anterior third; metapleuron coriaceous with shallow medium-sized punctures, more sparsely distributed than on mesopleuron.

**Propodeum:** ATC strong, strongly arched along ASu (so anterior margin of ASu strongly convex); PTC obsolete in centre, strong at intersection with MLC and for a short distance along AD, otherwise obsolete along AD, very strong along AJC; MLC obsolete and slightly convergent along ASu, obsolete (represented by wrinkles) and strongly convergent along AP; LLC present along AJC, very weakly represented at intersection with ATC, otherwise absent; PC strong, not connected to spiracle; spiracular area sloping, coriaceous with small shallow punctures; posterior area weakly rugopunctate, becoming wrinkled apically.

**Wings:** Wing L: 14.0 mm, CI: 0.62, AI: 1.73, SDI: 1.29, ICI: 0.80, wing veins dark brown, stigma reddish-brown, fenestra restricted to area below stigma, trichiae slightly sparser below prestigma, ramellus long.

**Legs:** CL/CW: 2.0; FL/FW: 9.5, MT1/MT2: 2.1; MTS: 0.78.

**Metasoma:** Sides of petiole gradually expanding at spiracles.

**Colour:** Uniformly reddish-orange; stemmaticum distinctly darker, dark reddish-brown; palps and scutellum very slightly paler than base colour, tegula and extreme dorsal part of mesepimeron dark yellowish, mesopleural fovea slightly darker than base colour.

##### Seasonality.

The one collection record is from May 13.

#### Ophion dombroskii

Taxon classificationAnimaliaHymenopteraIchneumonidae

Schwarzfeld sp. n.

http://zoobank.org/E1B81D98-14D3-4B7B-959B-01E9873B93DA

**Plate 7.**
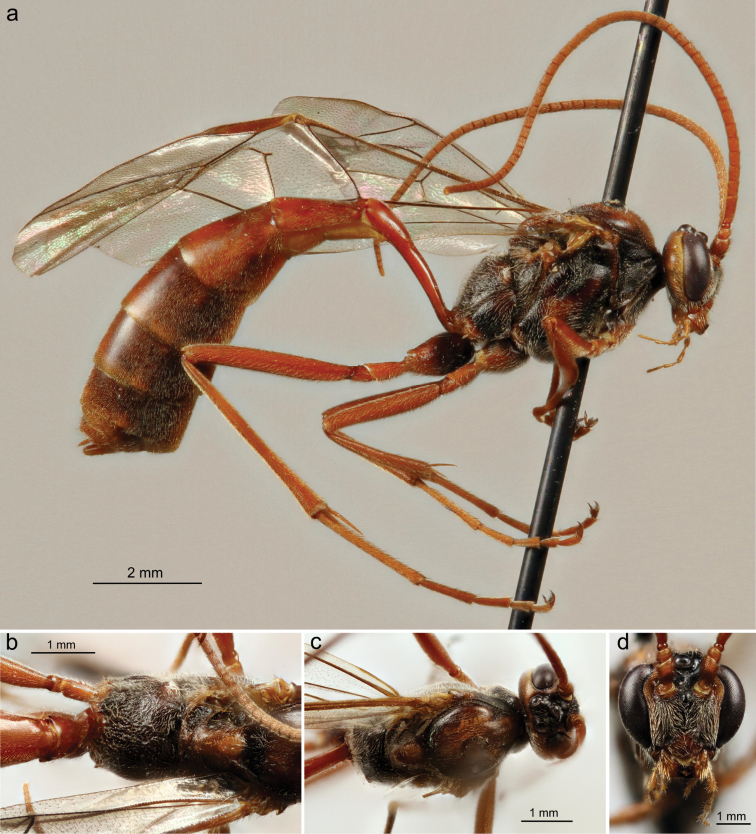
*Ophion dombroskii* Schwarzfeld sp.n., type specimen; **a** habitus **b** propodeum and scutellum **c** mesothorax and head **d** face.

Figures 10g , 11g ; Plate 7

##### Type material.

Holotype ♀ (MS13975, DNA6548, GenBank KF594760, KF615971, KF616341) CAN: SK: nr. Newton L., 49.301 -107.764, 20 v 2011, UV trap, J. Dombroskie (CNC).

##### Etymology.

This species is named for Jason Dombroskie, who was kind enough to collect the only known specimen of this species on an otherwise rainy and utterly unsuccessful moth-collecting trip.

##### Diagnosis.

Wing L: 10.8 mm, Flag: 51; The head and thorax of this species are almost entirely black, making this species easily recognizable. It also has unusually short antennal segments, widely separated ocelli and a long, narrow scutellum.

##### Description.

**Head:** Eyes slightly convergent in frontal view; stemmaticum slightly raised, sulci surrounding stemmaticum complete; IOD/OL: 1.20, OOD/OL: 0.45; occipital carina rounded, OC/OL: 1.20; temple receding, approximately equal to width of eye in lateral view; clypeus 0.5× as high as apical width, coriaceous, punctures irregularly sized (coarse to very small) and sparsely, irregularly distributed, denser on sides, CH/CAW: 0.50; face with medium-sized punctures, approximately separated by their diameter and connected with strong microreticulation, smaller on sides of face; FW/FH: 1.34; antennae short, 51 flagellomeres, F1: 2.7, F20: 1.1; MS/MW: 0.6; GI/MW: 0.8.

**Mesosoma:** Mesoscutum coriaceous, evenly punctate with minute punctures, separated by several times their diameter; mesopleuron and metapleuron strongly coriaceous, evenly, coarsely punctate with punctures separated by their diameter or less; mesopleuron above mesopleural fovea subpolished, punctures minute, separated by 2-3× their diameter; epicnemial carina pleurosternal angle obtuse; scutellum with lateral carina strong along anterior half, SL/SW: 1.8.

**Propodeum:** ATC strong and strongly arched along ASu (so anterior margin of ASu strongly convex), weak to obsolete along AD; PTC mostly obsolete, represented by wrinkles, forming small crests where intersects with MLC, strong along AJC, especially strong at propodeal apophysis; MLC obsolete, faintly represented by wrinkles along ASu and even more indistinctly along AP; LLC present as a wrinkle along AJC, otherwise absent; PC strong, not connected to spiracle; spiracular area sloping, coriaceous with numerous small punctures; posterior area weakly wrinkled and punctate, more wrinkled apically.

**Wings:** Wing L=10.8 mm, CI: 0.49, AI: 1.39, SDI: 1.15, ICI: 0.83; wings with veins dark brown, stigma light brown, fenestra not extending below prestigma, ramellus short.

**Legs:** CL/CW: 1.5, FL/FW: 7.2, MT1/MT2: 3; MTS: 0.85.

**Colour:** Head: black; orbits, temple, vertex, mandibles, palps and clypeus except for extreme base reddish-orange; Mesosoma: black; mesocutum (except margins and base of notauli), scutellum, anterior margin and an indistinct area in the centre of mesopleuron, apical margin of propodeum, apical half of coxae and legs reddish-orange; mesepimeron reddish-orange ventrally and yellowish dorsally; metasoma reddish-orange; ovipositor sheath same colour as metasoma.

##### Seasonality.

The one collection record is from May 20.

##### Remarks.

This species is unusual because of its extensive black markings. The unusually short antennae and black markings indicate that this species may be diurnally active (Gauld 1980). Gauld (1985) mentions a few undescribed deserticolous species with short antennae and quadrate central flagellomeres; we have not seen these specimens, so it is unknown whether this species should be considered among them.

## Supplementary Material

XML Treatment for Ophion keala

XML Treatment for Ophion idoneus

XML Treatment for Ophion importunus

XML Treatment for Ophion clave

XML Treatment for Ophion aureus

XML Treatment for Ophion brevipunctatus

XML Treatment for Ophion dombroskii

## Appendix 1

**Appendix 1:** List of all specimens included in molecular and morphometric analyses.

Accession^1^	Provenance^2^	Lat-long	Date	Coll.^3^	Species^4^		GenBank		
						Sex	COI	ITS2	28S
***Ophion scutellaris* species group**									
DNA3970	AB: Machesis Lake PRA	58.325 -116.578	10 vi 2008	DM	*aureus*^C,G^	F		KF615968	
DNA3911	AB: nr. Tangent Park	56.092 -117.542	7 v 2008	DM	*aureus*^C,G^	M	KF594487	KF615965	KF616311
DNA3961	AB: nr. Tangent Park	56.092 -117.542	7 v 2008	DM	*aureus*^C,G^	M	KF594535	KF615966	
DNA3975	AB: nr. Tangent Park	56.092 -117.542	7 v 2008	DM	*aureus*^C,G^	M		KF615969	
MS2313	AB: nr. Tangent Park	56.092 -117.542	7 v 2008	DM	*aureus*^C,G^	M			
MS2315	AB: nr. Tangent Park	56.092 -117.542	7 v 2008	DM	*aureus*^C,G^	M			
MS2316	AB: nr. Tangent Park	56.092 -117.542	7 v 2008	DM	*aureus*^C,G^	M			
MS2314	AB: nr. Tangent Park	56.092 -117.542	7 v 2008	DM	*aureus*^C,G^	M			
MS2311	AB: nr. Tangent Park	56.092 -117.542	7 v 2008	DM	*aureus*^C,G^	M			
MS2320	AB: nr. Tangent Park	56.092 -117.542	7 v 2008	DM	*aureus*^C,G^	M			
DNA3939	ON: nr. Carp	45.385 -76.008	13 v 2008	BCS	*brevipunctatus*^C,G^	F	KF594513	KF615967	KF616314
DNA7381	AB: Jenner	50.844 -111.154	2 vi 2010	GGA	*clave*^C,G^	M	KF594961		
DNA7382	AB: Jenner	50.842 -111.151	09 v 2007	MDS	*clave*^C,G^	M	KF594962	KF615970	
DNA7383	AB: Jenner	50.844 -111.154	2 vi 2010	GGA	*clave*^C,G^	F	KF594963		
DNA7391	AB: Jenner	50.842 -111.151	07 vi 2007	GGA	*clave*^C,G^	F	KF594970		
MS72	AB: Jenner	50.842 -111.151	9 v 2007	MDS	*clave*^C,G^	F			
MS1434	AB: Jenner	50.842 -111.151	26 v 2007	JJD	*clave*^C,G^	F			
MS13752	AB: Jenner	50.844 -111.154	2 vi 2010	GGA	*clave*^C,G^	F			
MS13748	AB: Jenner	50.844 -111.154	2 vi 2010	GGA	*clave*^C,G^	F			
DNA6944	AB: Spruce Grove	53.4 -113.9	28 v–2 vi 1989	ATF	*clave*^C,G^	F	KF594831	KF615972	KF616352
MS12198	AB: Spruce Grove	53.4 -113.9	28 v–2 vi 1989	ATF	*clave*^C,G^	F			
MS12199	AB: Spruce Grove	53.4 -113.9	28 v–2 vi 1989	ATF	*clave*^C,G^	F			
MS12386	AB: Stettler Co.	52.154 -112.713	26 v 2010	CDB	*clave*^C,G^	F			
MS11578	AB: Stettler Co.	52.154 -112.713	24 v 2009	CDB	*clave*^C,G^	M			
n/a	MB: Brandon	49.83 -99.96	29 v 1949	?	*clave*^C^	F			
n/a	ON: Cambridge	43.46 -80.52	18–21 v 1992	S&C	*clave*^C^	F			
MS10758	ON: Leeds	44.487 -76.008	27 iv 2009	BCS	*clave*^C,G^	F			
MS10763	ON: Leeds	44.487 -76.008	27 iv 2009	BCS	*clave*^C,G^	F			
DNA6548	SK: nr. Newton Lake	49.301 -107.764	20 v 2011	JJD	*dombroskii*^C,G^	F	KF594760	KF615971	KF616341
ENT008-002252	BC: Robson	49.34 -117.70	17 v 1954	HRF	*idoneus*^C^	F			
ENT008-002250	BC: Robson	49.34 -117.70	15 v 1951	HRF	*idoneus*^C^	F			
ENT008-005274	BC: Robson	49.34 -117.70	14 v 1954	HRF	*idoneus*^C^	M			
ENT008-002230	BC: Robson	49.34 -117.70	29 v 1953	HRF	*idoneus*^C^	M			
DNA6551	AB: 48 km NW Dixonville	56.753 -118.328	28 v–10 vi 2008	MDS	*idoneus*^C,G^	F	KF594763	KF615949	
MS7373	AB: Bragg Cr.	50.917 -114.533	15 viii 2007	FAHS	*idoneus*^C,G^	F			
MS4026	AB: Calgary	51.115 -114.142	18 v 2010	TP	*idoneus*^C,G^	F			
DNA3908	AB: Edmonton	53.521 -113.521	20 v 2008	JJD	*idoneus*^C,G^	F	KF594484	KF615962	
MS100	AB: Edmonton	53.527 -113.519	28 v 2007	MDS	*idoneus*^C,G^	M			
MS107	AB: Edmonton	53.527 -113.519	28 v 2007	MDS	*idoneus*^C,G^	M			
MS103	AB: Edmonton	53.527 -113.519	28 v 2007	MDS	*idoneus*^G^	M			
DNA3933	AB: Edmonton	53.545 -113.439	15 vi 2008	GGA	*idoneus*^C,G^	M	KF594507	KF615951	
DNA3910	AB: Edmonton	53.545 -113.439	15–16 v 2008	GGA	*idoneus*^C,G^	F	KF594486	KF615953	
MS2274	AB: Edmonton	53.545 -113.439	15–16 v 2008	GGA	*idoneus*^G^	F			
MS2280	AB: Edmonton	53.545 -113.439	15–16 v 2008	GGA	*idoneus*^G^	F			
MS2284	AB: Edmonton	53.545 -113.439	17 v 2008	GGA	*idoneus*^G^	M			
DNA3974	AB: Edmonton	53.545 -113.439	23 v 2009	GGA	*idoneus*^C,G^	F	KF594547	KF615956	
MS2286	AB: Edmonton	53.545 -113.439	24 v 2008	GGA	*idoneus*^G^	F			
MS12169	AB: Edmonton	53.545 -113.439	31 v 2009	GGA	*idoneus*^G^	F			
MS12171	AB: Edmonton	53.545 -113.439	31 v 2009	GGA	*idoneus*^G^	F			
MS12172	AB: Edmonton	53.545 -113.439	31 v 2009	GGA	*idoneus*^G^	F			
MS78	AB: Edmonton	53.545 -113.439	v 2007	GGA	*idoneus*^C,G^	M			
MS80	AB: Edmonton	53.545 -113.439	v 2007	GGA	*idoneus*^G^	M			
MS12420	AB: Erskine	52.322 -112.883	19 v 2010	CDB	*idoneus*^C,G^	F			
MS9764	AB: George L	53.953 -114.120	29 v 2007	MDS	*idoneus*^G^	F			
MS9763	AB: George L	53.953 -114.120	29 v 2007	MDS	*idoneus*^G^	M			
MS39	AB: George L	53.957 -114.125	25 v 2007	MDS	*idoneus*^G^	M			
MS11824	AB: George L	53.957 -114.125	25–29 v 2007	MDS	*idoneus*^G^	F			
MS11825	AB: George L	53.957 -114.125	25–29 v 2007	MDS	*idoneus*^G^	F			
MS11826	AB: George L	53.957 -114.125	25–29 v 2007	MDS	*idoneus*^G^	F			
MS11827	AB: George L	53.957 -114.125	25–29 v 2007	MDS	*idoneus*^G^	F			
MS11828	AB: George L	53.957 -114.125	25–29 v 2007	MDS	*idoneus*^G^	M			
MS13334	AB: George L	53.957 -114.125	29 v–6 vi 2007	MDS	*idoneus*^C,G^	M			
MS13333	AB: George L	53.957 -114.125	29 v–6 vi 2007	MDS	*idoneus*^C,G^	M			
MS13319	AB: George L	53.957 -114.125	29 v–6 vi 2007	MDS	*idoneus*^G^	M			
MS13330	AB: George L	53.957 -114.125	29 v–6 vi 2007	MDS	*idoneus*^G^	M			
MS13331	AB: George L	53.957 -114.125	29 v–6 vi 2007	MDS	*idoneus*^G^	M			
MS13332	AB: George L	53.957 -114.125	29 v–6 vi 2007	MDS	*idoneus*^G^	M			
MS13336	AB: George L	53.957 -114.125	29 v–6 vi 2007	MDS	*idoneus*^C,G^	F			
MS13320	AB: George L	53.957 -114.125	29 v–6 vi 2007	MDS	*idoneus*^G^	F			
MS13322	AB: George L	53.957 -114.125	29 v–6 vi 2007	MDS	*idoneus*^G^	F			
MS13324	AB: George L	53.957 -114.125	29 v–6 vi 2007	MDS	*idoneus*^G^	F			
MS13325	AB: George L	53.957 -114.125	29 v–6 vi 2007	MDS	*idoneus*^G^	F			
MS13326	AB: George L	53.957 -114.125	29 v–6 vi 2007	MDS	*idoneus*^G^	F			
MS13328	AB: George L	53.957 -114.125	29 v–6 vi 2007	MDS	*idoneus*^G^	F			
MS13329	AB: George L	53.957 -114.125	29 v–6 vi 2007	MDS	*idoneus*^G^	F			
MS13335	AB: George L	53.957 -114.125	29 v–6 vi 2007	MDS	*idoneus*^G^	F			
MS13317	AB: George L	53.957 -114.125	29 v–6 vi 2007	MDS	*idoneus*^G^	F			
MS12920	AB: George L	53.957 -114.128	29 v–6 vi 2007	MDS	*idoneus*^G^	M			
MS12922	AB: George L	53.957 -114.128	29 v–6 vi 2007	MDS	*idoneus*^G^	M			
MS12924	AB: George L	53.957 -114.128	29 v–6 vi 2007	MDS	*idoneus*^G^	M			
MS12926	AB: George L	53.957 -114.128	29 v–6 vi 2007	MDS	*idoneus*^G^	M			
MS12927	AB: George L	53.957 -114.128	29 v–6 vi 2007	MDS	*idoneus*^G^	M			
MS12928	AB: George L	53.957 -114.128	29 v–6 vi 2007	MDS	*idoneus*^G^	M			
MS12923	AB: George L	53.957 -114.128	29 v–6 vi 2007	MDS	*idoneus*^G^	F			
MS12925	AB: George L	53.957 -114.128	29 v–6 vi 2007	MDS	*idoneus*^G^	F			
n/a	AB: Pigeon Lake	53.072 -114.072	38 v 2006	FAHS	*idoneus*^C,G^	M			
DNA3977	AB: Pigeon Lake	53.072 -114.072	3 vi 2008	FAHS	*idoneus*^C,G^	F	KF594549	KF615957	
DNA3950	AB: Pigeon Lake	53.072 -114.072	3 vi 2008	FAHS	*idoneus*^C,G^	F	KF594524	KF615952	
MS12475	AB: Rochon Sands PP	52.46 -112.88	2 vi 2010	CDB	*idoneus*^C,G^	F			
MS12491	AB: Rochon Sands PP	52.46 -112.88	2 vi 2010	CDB	*idoneus*^C,G^	M			
MS2261	AB: Sherwood Park	53.478 -113.229	12–15 v 2008	GRP	*idoneus*^G^	F			
MS12197	AB: Spruce Grove	53.4 -113.9	28 v–2 vi 1989	ATF	*idoneus*^C,G^	M			
MS12444	AB: Winfield	53.01 -114.50	12 vi 2010	CDB	*idoneus*^C,G^	F			
MS12434	AB: Winfield	53.01 -114.50	12 vi 2010	CDB	*idoneus*^G^	F			
MS12433	AB: Winfield	53.01 -114.50	12 vi 2010	CDB	*idoneus*^G^	F			
MS12435	AB: Winfield	53.01 -114.50	12 vi 2010	CDB	*idoneus*^G^	F			
MS12335	AB: Winfield	53.01 -114.50	15 v 2010	CDB	*idoneus*^C,G^	M			
MS11457	AB: Winfield	53.01 -114.50	16 v 2009	CDB	*idoneus*^G^	M			
	MB: Brandon	49.83 -99.96	29 v 1949	?	*idoneus*^C^	F			
	MB: Winnipeg	49.74 -97.13	1 vi 1973	CS	*idoneus*^C^	F			
MS2933	ON: Bells Corners	45.272 -75.808	18 v 2010	BCS	*idoneus*^C,G^	F			
MS2927	ON: Bells Corners	45.272 -75.808	18 v 2010	BCS	*idoneus*^C,G^	M			
MS2932	ON: Bells Corners	45.272 -75.808	18 v 2010	BCS	*idoneus*^C,G^	M			
DNA5554	ON: Grenadier Is.	44.4 -75.9	10–21 vi 1994	CNC	*idoneus*	?	KF594578	KF615955	KF616320
DNA6552	ON: Grenadier Is.	44.4 -75.9	24 v–9 vi 1994	CNC	*idoneus*^C,G^	M	KF594764	KF615960	
DNA3976	ON: Leeds	44.487 -76.008	7 vi 2008	BCS	*idoneus*^C,G^	F	KF594548	KF615961	
MS10780	ON: Ottawa	45.356 -75.707	5 v–5 vi 2008	HG	*idoneus*^C,G^	M			
DNA5551	ON: Ottawa	45.356 -75.707	5 v–5 vi 2008	HG	*idoneus*^C,G^	M	KF594575	KF615954	
DNA5552	ON: Ottawa	45.356 -75.707	5 v–5 vi 2008	HG	*idoneus*^C,G^	F	KF594576	KF615958	
DNA5717	ON: Ottawa	45.356 -75.707	5 v–5 vi 2008	HG	*idoneus*^C,G^	F	KF594635	KF615959	
n/a	SK: Saskatoon	52.1 -106.6	22 v 1940	DRF	*idoneus*^C^	F			
DNA6907	AB: 8 km NW Winfield	53.01 -114.50	15 v 2010	CDB	*importunus*^C,G^	F	KF594814	KF615963	KF616346
MS13904	AB: 8 km NW Winfield	53.01 -114.50	15 v 2010	CDB	*importunus*^C,G^	F			
MS12153	ON: Bells Corners	45.295 -75.830	3 v 2010	BCS	*importunus*^C,G^	F			
MS7746	AB: 54 km NW Dixonville	56.86 -118.31	11 vi 2007	BBB	*keala*^C,G^	F			
DNA7324	AB: 62 km WNW Dixonville	56.684 -118.644	7 vi 2008	BBB	*keala*^C,G^	F	KF594914	KF615944	
DNA7327	AB: 62 km WNW Dixonville	56.685 -118.641	26 v 2008	BBB	*keala*^C,G^	F	KF594917	KF615946	
DNA3904	AB: Porcupine Hills	49.972 -114.087	28 v 2008	JJD	*keala*^C,G^	M	KF594480	KF615945	KF616307
DNA3960	AB: Porcupine Hills	49.972 -114.087	28 v 2008	JJD	*keala*^C,G^	F	KF594534	KF615950	
DNA3965	AB: Porcupine Hills	49.972 -114.087	29 v 2008	JJD	*keala*^C,G^	F	KF594539	KF615948	
DNA3980	AB: Porcupine Hills	49.972 -114.087	29 v 2008	JJD	*keala*^C,G^	F	KF594552	KF615947	
DNA6515	AB: Porcupine Hills	49.972 -114.087	15 vi 2009	JJD	*keala*^C,G^	F	KF594730	KF615943	KF616332
MS2237	AB: Porcupine Hills	49.972 -114.087	28 v 2008	JJD	*keala*^C,G^	F			
MS11464	AB: Winfield	53.01 -114.50	16 v 2009	CDB	*A*^ C,G^	F			
MS4024	AB: Calgary	51.115 -114.142	18 v 2010	TP	*B*^C,G^	F			
MS4310	AB: Beaver Mines Lake	49.371 -114.295	16 vii 2009	TP	*C*^C,G^	F			
MS2322	AB: nr. Tangent Park	56.092 -117.542	7 v 2008	DM	*D*^C,G^	M			
MS11823	AB: George Lake	53.957 -114.125	25–29 v 2007	MDS	*E*^C,G^	M			
n/a	UK	not listed	not listed		*scutellaris*				EU378720
DNA7524	UK	?	?	GRB	*scutellaris A*		KF595042	KM982696	
DNA6914	UK: Pitstone Commons	51.81 -0.593	22 iv–7 v 2011	GRB	*scutellaris A*		KF594819	KF615964	KF616305
DNA7515	UK: Radnage	51.659 -0.858	3 v 2008	AMG	*scutellaris A*		KF594993		
DNA7558	UK: Radnage	51.659 -0.858	21 vii 2011	AMG	*scutellaris A*		KF595030		
DNA7519	UK: Westcott	51.848 -0.962	20 iii 2009	DW	*scutellaris B*		KF594995		
**Not *Ophion scutellaris* species group**									
DNA6501	AB: 6 km N Guy	55.607 -117.161	22 vii–16 viii	BAM	*Ophion* sp.		KF594717	KF616256	KF616362
DNA6913	UK: Aldbury	51.799 -0.601	9 v 2011	GRB	*minutus*		KF594818	KF616300	KF616360
DNA3902	AB: Kootenay Plains PRA	52.064 -116.422	14 vii 2007	MDS	*Ophion* sp.		KF594478	KF615984	KF616308
DNA6550	SK: nr. Newton Lake	49.301 -107.764	20 v 2011	JJD	*Ophion* sp.		KF594762	KF616150	KF616343
DNA5559	BC: Vancouver	49.2 -123.2	3–17 ix 1997	IK	*Ophion* sp.		KF594583	KF615978	KF616371
DNA5742	BC: Glacier NP	51.26 -117.57	8 vii 2010	MDS	*Ophion* sp.		KF594660	KF615911	KF616337
DNA3957	AB: 21 km S Whitelaw	55.928 -117.995	29 vi 2008	DM	*Ophion* sp.		KF594531	KF615930	KF616381
DNA5566	MB: Winnipegosis	51.651 -99.945	16 vi 2007	HG	*Ophion* sp.		KF594589	KF616242	KF616321
DNA3901	AB: Kootenay Plains PRA	52.064 -116.422	14 vii 2007	MDS	*Enicospilus* sp.		KF594477		KF594477

1. **Specimen accession numbers:** DNA = Sperling lab (University of Alberta) DNA voucher number for newly sequenced specimens; MS = M. Schwarzfeld specimen number; ENT = RBCM database number

2. **Abbreviations:** NP = National Park; PP = Provincial Park; PRA = Provincial Recreation Area

3. **Collectors:** AMG = A.M. George; ATF = A.T. Finnamore; BAM = B.A. Mori; BBB = B.B. Bodeux; BCS = B.C. Schmidt; CDB = C.D. Bird; CNC = Canadian National Collection Hym team; CS = C. Starr; DM = D. Macaulay; DRF = D.R. Foskell; DW = D. Wilton; FAHS = F.A.H. Sperling; GGA = G.G. Anweiler; GRB = G.R. Broad; HG = H. Goulet; HRF = H.R. Foxlee; IK = I. Klimeszewski; JJD = J.J. Dombroskie; MDS = M.D. Schwarzfeld; S&C = Skevington & Cannings; TP = T. Pike<br/> **4. Species:** All Nearctic species were determined by MDS; all newly sequenced Palearctic species were determined by GRB; C = specimen included in classical morphometric study; G = specimen included in geometric morphometric study

4. **Species:** All Nearctic species were determined by MDS; all newly sequenced Palearctic species were determined by GRB; C = specimen included in classical morphometric study; G = specimen included in geometric morphometric study
